# Durlobactam to boost the clinical utility of standard of care β-lactams against *Mycobacterium abscessus* lung disease

**DOI:** 10.1128/aac.01046-24

**Published:** 2024-11-20

**Authors:** Dereje A. Negatu, Wassihun Wedajo Aragaw, Tewodros T. Gebresilase, Sindhuja Paruchuri, Firat Kaya, Sung Jae Shin, Peter Sander, Véronique Dartois, Thomas Dick

**Affiliations:** 1 Center for Discovery and Innovation, Hackensack Meridian Health, Nutley, New Jersey, USA; 2 Center for Innovative Drug Development and Therapeutic Trials for Africa (CDT-Africa), Addis Ababa University 37602 , Addis Ababa, Ethiopia; 3 Armauer Hansen Research Institute 70605 , Addis Ababa, Ethiopia; 4 Institute of Biotechnology, Addis Ababa University 37602 , Addis Ababa, Ethiopia; 5 Department of Microbiology, Graduate School of Medical Science, Brain Korea 21 Project, Yonsei University College of Medicine 37991 , Seodaemun-gu, Seoul, South Korea; 6 Institut für Medizinische Mikrobiologie, Universitat Zurich Institut fur Medizinische Mikrobiologie 30990 , Zürich, Switzerland; 7 National Reference Center for Mycobacteria, Universitat Zurich Institut fur Medizinische Mikrobiologie 30990 , Zürich, Switzerland; 8 Department of Medical Sciences, Hackensack Meridian School of Medicine 576909 , Nutley, New Jersey, USA; 9 Department of Microbiology and Immunology, Georgetown University 8368 , Washington, DC, USA; Bill & Melinda Gates Medical Research Institute, Cambridge, Massachusetts, USA

**Keywords:** β-lactams, *Mycobacterium abscessus*, lung infection, MspA, MmpL11, RshA, drug resistance

## Abstract

β-Lactams present several desirable pharmacodynamic features leading to the rapid eradication of many bacterial pathogens. Imipenem (IPM) and cefoxitin (FOX) are injectable β-lactams recommended during the intensive treatment phase of pulmonary infections caused by *Mycobacterium abscessus* (Mab). However, their potency against Mab is many-fold lower than against Gram-positive and Gram-negative pathogens for which they were optimized, putting into question their clinical utility. Here, we show that adding the recently approved durlobactam-sulbactam (DUR-SUL) pair to either IPM or FOX achieves growth inhibition, bactericidal, and cytolytic activity at concentrations that are within those achieved in patients and below the clinical breakpoints established for each agent. Synergies between DUR-SUL and IPM or FOX were confirmed across a large panel of clinical isolates. Through *in vitro* resistant mutant selection, we also show that adding DUR-SUL abrogates acquired resistance to IPM and FOX. Since the use of β-lactam injectables is firmly grounded in clinical practice during the intensive treatment phase of Mab pulmonary disease, their potentiation by FDA-approved DUR-SUL to bring minimum inhibitory concentration distributions within achievable concentration ranges could offer significant short-term benefits to patients, while novel β-lactam combinations are optimized specifically against Mab pulmonary infections, for which no reliable cure exists.

## INTRODUCTION

*Mycobacterium abscessus* pulmonary disease (Mab-PD) is treated for many months to years with multiple antibiotics until sputum cultures remain negative for 12 months. Yet cure rates are poor, around 50% across patient populations (1). One root cause of such dismal treatment performance is that antibiotics available to clinicians were repurposed from other infectious diseases rather than optimized to eradicate Mab, while Mab is intrinsically resistant to many drug classes (2). Guidelines suggest a biphasic approach: an initial 3- to 12-week intensive phase includes one to three parenteral agents to be selected among amikacin, imipenem (IPM) or cefoxitin (FOX), and tigecycline, and is followed by a continuation phase with oral and inhaled agents. The optimal duration of the intensive phase is unknown and a positive impact of longer duration on treatment outcome has not been established (3). The confidence in the estimates of effect of the two β-lactam injectables, IPM and FOX, is low due to the lack of association between susceptibility category (susceptible, intermediate, or resistant) and culture conversion or microbiological cure (4). In a hollow fiber model, the intensive phase standard of care, including injectables amikacin and FOX combined with oral clarithromycin, failed to reduce the initial inoculum, and resulted in emergence of resistance to FOX after 14 days despite the three-drug treatment and despite FOX exposures being higher than achieved in most patients receiving standard doses (5).

β-Lactam injectables, like many antibiotics recommended against Mab-PD, do not achieve therapeutic concentrations at tolerated doses upon long-term treatment. Against Gram-positive and Gram-negative moderate and severe infections, IPM and FOX are infused three or four times daily (6, 7), but given the multi-week to month duration of the intensive treatment phase of Mab-PD, this is often reduced to twice daily for operational feasibility (8). In addition, evidence-based clinical breakpoints are lacking (9), and those proposed by the Clinical and Laboratory Standards Institute (CLSI) and European Committee on Antimicrobial Susceptibility Testing (EUCAST) against Mab for IPM and FOX (https://www.eucast.org/ast_of_mycobacteria [10]) are higher than against other infections (https://www.fda.gov/media/92766/download [11, 12]). Probability of target attainment calculated for patients under optimized dosing schedules, to treat infections with less complex disease pathology and markedly higher susceptibility to IPM or FOX than Mab (6, 7, 13, 14), provide a compelling explanation for the poor clinical performance of IPM and FOX in Mab-PD. A pilot study that measured the steady-state concentrations of FOX in patients with Mab-PD predicted that a continuous infusion of at least 6 g in 24 h is required to achieve effective concentrations, assuming MIC ≤ 16 μg/mL (15). But FOX infused only twice daily to treat Mab-PD is frequently discontinued due to neutropenia and thrombocytopenia (16).

Yet β-lactams present many attractive features, providing a strong incentive to restore their clinical utility against Mab-PD. They are among the oldest antibiotics in medical practice. Their pharmacokinetic and pharmacodynamic (PK-PD) properties are well understood (17). Owing to their lytic mechanism of action, they cause irreversible damage to the structural integrity of the cell and are bactericidal around their minimum inhibitory concentration (MIC) against most pathogens (18) including mycobacteria (19–21), a desirable and uncommon property of anti-Mab agents. They are also bactericidal to non-replicating drug-tolerant Mab (22), a finding that could be attributed to their targeting of peptidoglycan remodeling that occurs in the non-replicating state in *Mycobacterium tuberculosis* (23). Given the functional redundancy of penicillin-binding proteins (PBPs) and peptidoglycan synthesis (PG) enzymes (24, 25), canonical target-based mutations conferring phenotypic resistance are uncommon, another favorable property of the β-lactam class.

Although IPM and FOX are exclusively intravenous agents, they are firmly grounded in clinical practice during the intensive treatment phase of Mab-PD. Therefore, potentiation by FDA-approved agents to bring MIC distributions within achievable concentration ranges could offer significant short-term benefits to patients. Bla_Mab_ is Mab’s major β-lactamase (26), responsible for inactivating several β-lactams, and is effectively blocked by avibactam (27) but not clavulanate (28). Genetic inactivation of Bla_Mab_, however, has little impact on the potency of IPM or FOX (26). Likewise, a screen of FDA-approved oral and parenteral β-lactams with the β-lactamase inhibitors (BLI) relebactam, zidebactam, nacubactam (diazabicyclooctanes [DBOs]), or vaborbactam (a boronic acid BLI) showed minor twofold improvements of IPM potency and no impact on FOX (29–31), consistent with FOX being weakly hydrolyzed *in vitro* by Bla_Mab_ with a very low catalytic efficiency (28). Thus, a potentiation approach strictly relying on BLIs is unlikely to deliver a drastic potency increase for IPM and FOX.

The durlobactam-sulbactam (DUR-SUL) injectable combination was approved by the FDA in May 2023 for the treatment of bacterial pneumonia caused by carbapenem-resistant *Acinetobacter baumannii*, as a co-formulation. Bonomo and coll. have shown that DUR, a DBO BLI, is an inhibitor of Bla_Mab_ and protects Bla_Mab_ substrates against hydrolysis, but also exhibits intrinsic activity through inhibition of L,D-transpeptidases (32). Little is known about the potential role of SUL against Mab, which does not inhibit Bla_Mab_ (28). In a comprehensive PBP occupancy study, SUL inactivated PonA2 and bound PbpA at 2 and 16 μg/mL, respectively (33).

Against *A. baumannii*, SUL inhibits peptidoglycan biosynthesis by targeting two major PBPs, and DUR protects SUL as the BLI, inhibiting Ambler class A, C, and D serine β-lactamases (34). Like other DBO, DUR uses a reversible mechanism of inhibition through β-lactamase active site carbamoylation and dissociates intact from the β-lactamase, rather than being released as a hydrolysis product, remaining available to inhibit another enzyme molecule (35).

Since dual β-lactam and β-lactam/BLI approaches increasingly emerge as a promising treatment strategy against Mab-PD (36, 37), we systematically investigated the quantitative impact of DUR-SUL on the potency and bactericidal activity of IPM or FOX, and the extent to which their clinical utility could be improved. We also measured the effect of added DUR-SUL on the frequency of resistance to IPM or FOX and identified genetic determinants of resistance to single and combination injectables. Our results indicate that the DUR-SUL pair brings the MIC distribution of both IPM and FOX below the proposed susceptibility breakpoints and shows promise for direct advancement into clinical trials or clinical use for the treatment of Mab lung infections.

## RESULTS

### DUR significantly enhances the potency of IPM and FOX against *M. abscessus*

We first screened a comprehensive set of broad-spectrum BLIs belonging to the three major structural classes (38), which are either approved or in late clinical development (Fig. S1A; Table S1), alone and in combination with IPM and FOX. Our objectives were to (i) determine whether any of them not only acts as a BLI but also inhibits PBP involved PG synthesis, as seen for DUR in Mab and additional BLIs against other bacteria (39); (ii) confirm the drastic potentiation of IPM by DUR and determine if it extends to FOX; and (iii) determine whether any other BLI enhances the potency of IPM or FOX to the same or higher extent than DUR. To mitigate the relative instability of β-lactams (40), the MIC assay was carried out in Middlebrook 7H9, in which Mab strains grow faster than in cation-adjusted Mueller-Hinton broth (CAMHB), allowing for OD_600_ reading after 3 days instead of 5, as previously optimized (29). DUR and SUL were significantly more stable than earlier generation β-lactams with half-lives of 65 h and >120 h in Middlebrook 7H9, respectively (Fig. S1B). SUL stability is consistent with published data (40).

**Fig 1:**
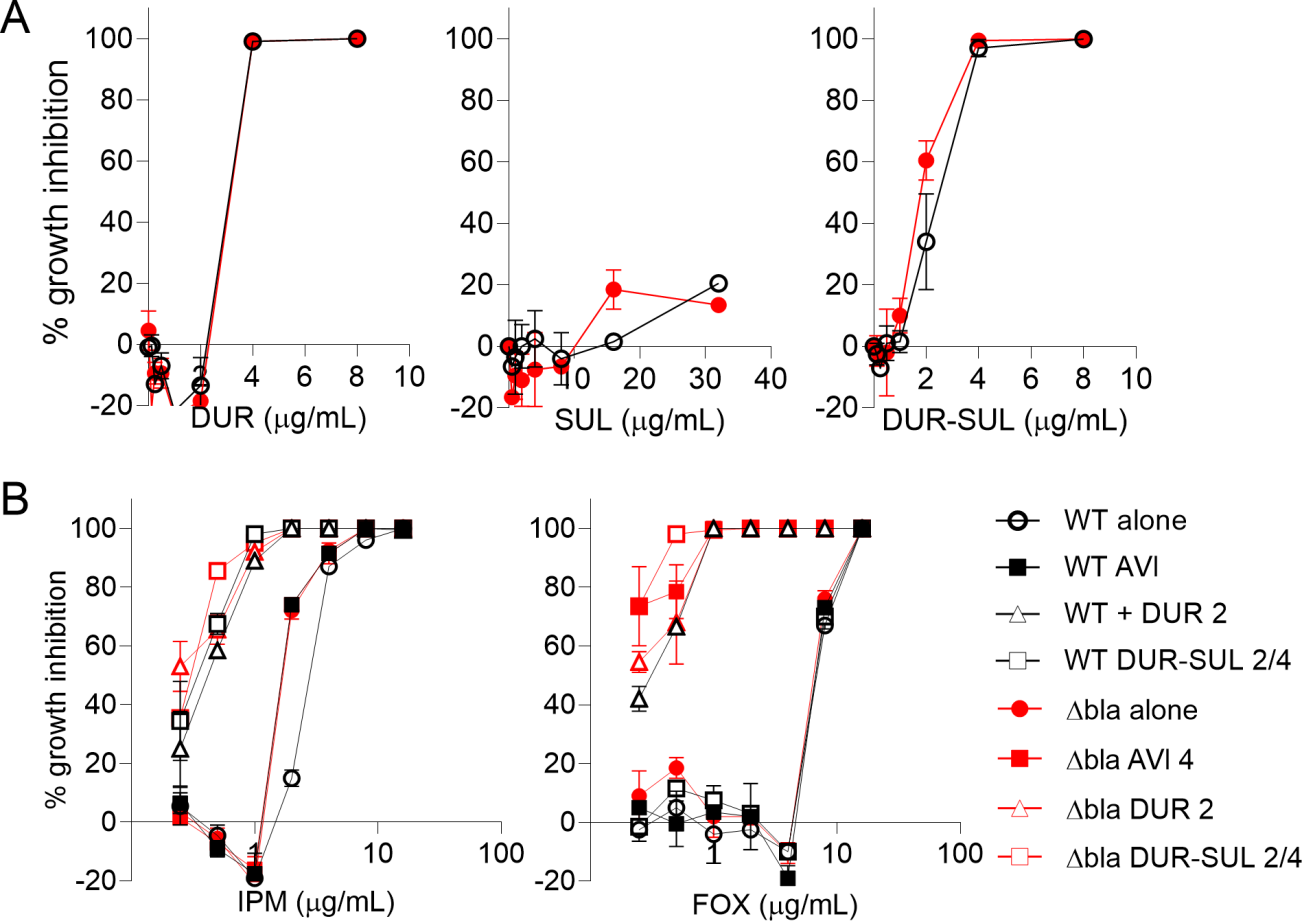
Impact of DUR on the growth inhibitory activity of IPM and FOX against Mab ATCC 19977. (**A**) Dose-response MIC of DUR, SUL, and DUR-SUL against Mab ATCC 19977 wild type and the isogenic Bla_Mab_ knockout (Δbla). (**B**) Dose-response growth inhibition of IPM and FOX with DUR fixed at 2 µg/mL and avibactam (AVI) at 4 µg/mL against Mab ATCC 19977. Percent growth inhibition was calculated relative to untreated control after subtracting partial growth inhibition due to DUR when relevant.

Among the 11 BLIs tested, only DUR inhibited Mab growth with an IC_90_ (concentration that inhibits 90% growth) of 10 μM or 4 μg/mL (IC_90_ >100 μM for all other BLIs, Table S1; Fig. 1A). Next, we combined each BLI with IPM or FOX at 10 μM side-by-side, against wild-type (WT) Mab ATCC 19977 and an isogenic ΔBla_Mab_ (26). Amoxicillin (AMX) was included as a positive control, given its known susceptibility to hydrolysis by Bla_Mab_. As expected, the potency of AMX increased in ΔBla_Mab_ compared to WT, and in the presence of all BLIs in WT Mab, as previously shown for a subset of these BLIs (29, 30, 32, 41). There was no further potentiation of AMX by the BLIs in ΔBla_Mab_, consistent with Bla_Mab_ being the major source of AMX hydrolysis, and the major target of the study BLIs (29, 30). In contrast, the potency of IPM and FOX was largely unaffected in ΔBla_Mab_ and only DUR substantially potentiated IPM and FOX in the WT background (Fig. S1C and D). This confirmed published negative findings for a subset of these BLIs (29, 30, 41), extended the observation to the rest of the set, indicating that a potentiation approach relying on BLIs (either FDA-approved or in the clinical development pipeline) does not deliver a therapeutically relevant potency increase for IPM and FOX. Of note, a dozen putative β-lactamase homologs have been detected in the Mab genome (24), supporting the hypothesis that one or more alternate β-lactamase(s) may be responsible for the limited potency of IPM and FOX against Mab. The results also confirmed the synergy between IPM and DUR (32) and extended it to FOX-DUR. In dose-response MIC assays, adding DUR at 5 μM (2 μg/mL sub-inhibitory concentration [42]) to IPM and FOX resulted in 6- and 14-fold reduction in IC_90_, respectively (Table S2), both in the WT and ΔBla_Mab_ backgrounds, consistent with DUR acting both as a BLI and PG synthesis inhibitor (32).

Since DUR is FDA-approved in combination with SUL, we focused on the pair for further in-depth biological profiling at clinically achieved concentrations, as it has the highest potential to boost IPM and/or FOX, standard of care β-lactams against Mab-PD. First, DUR at 2 μg/mL (Table S1) or 4 μg/mL (the CLSI susceptibility breakpoint for *A. baumannii* [42]) and SUL at 4 μg/mL (the CLSI susceptibility breakpoint [42]) were added to IPM or FOX in dose-response MIC assays, showing that DUR potentiates both IPM and FOX (the latter more strongly so than IPM) against Mab ATCC 19977, irrespective of the presence of a functional Bla_Mab_, and that SUL does not further enhance or negatively affect the positive interaction (Fig. 1B; Table 1). Checkerboard assays with the Mab-type strain further revealed a synergistic interaction between DUR or DUR-SUL and IPM or FOX, with fractional inhibitory concentration indices (FICIs) of 0.63 (additive) for the IPM/DUR-SUL combination and 0.38 (synergistic) for the FOX/DUR-SUL combination (Table S3). The positive impact of DUR and DUR-SUL on the growth inhibitory activity of IPM and FOX was conserved in the two other subspecies of the Mab complex, *M. abscessus* subsp. *massiliense* and *M. abscessus* subsp. *bolletii*, though they were generally slightly less susceptible to both IPM and FOX (Table 1), in line with prior findings (43). DUR’s published susceptibility breakpoint for lung infections caused by *A. baumanii* is 4 μg/mL. However, it exerts full growth inhibition of Mab ATCC 19977 at this concentration (Fig. 1A). Therefore, further potentiation experiments with fixed DUR concentrations were carried out at both 2 and 4 μg/mL, with and without 4 μg/mL SUL, and synergy/potentiation is reported at the lower 2 μg/mL concentration in the main tables and figures. Additional results with DUR supplemented at 4 μg/mL can be found in Supplemental Data set 1, as they are of clinical relevance. Across the three subspecies, adding DUR-SUL at 2/4 μg/mL sub-inhibitory concentrations resulted in an eightfold reduction of the visual MIC (MIC_vis_) of IPM and FOX (Table 1).

**TABLE 1:** Impact of DUR and DUR-SUL on the growth inhibitory activity of IPM and FOX against type strains of the *M. abscessus* complex^*a*^

Primary β-lactam	Potentiator [µg/mL]	Mab subsp. *abscessus* ATCC 19977			Mab subsp. *massiliense* CCUG 48898T			Mab subsp. *bolletii* CCUG 50184T		
		IC_50_	IC_90_	MIC_vis_	IC_50_	IC_90_	MIC_vis_	IC_50_	IC_90_	MIC_vis_
IPM	-	3	4	16	10	25	64	4	16	32
	DUR 2	0.7	1.5	2	2.5	6	16	1.5	4	8
	DUR-SUL 2/4	0.6	1.5	2	1.5	6	16	0.5	2	4
FOX	-	6	12	16	12	15	32	10	12	16
	DUR 2	0.7	1	2	0.8	2	4	0.7	1.5	2
	DUR-SUL 2/4	0.4	1	2	0.4	1.5	2	0.3	0.8	2
DUR	-	2.5	3	8	3.5	6	8	2.5	3.5	8
	IPM 1	0.25	1.5	4	2.5	5	8	1.5	3	4
	IPM 2	<0.02	0.13	2	0.5	3	4	<0.02	2	4
	IPM 4	<0.02	<0.02	0.5	<0.02	1.5	4	<0.02	0.6	2
	FOX 2	1.2	1.5	2	1.5	2	4	0.8	1.5	4
	FOX 4	0.3	0.4	1	0.5	0.8	1	0.3	0.4	1
	FOX 8	0.1	0.15	0.25	0.13	0.25	0.5	0.1	0.2	0.5
DUR-SUL	-	2.5	3	4	3	6	8	2.5	3.5	8
	IPM 1	0.06	1.5	2	2	3.5	8	0.8	2.5	4
	IPM 2	<0.02	0.016	1	0.13	2.5	4	<0.02	1.5	4
	IPM 4	<0.02	<0.02	0.5	<0.02	1	2	<0.02	0.5	2
	FOX 2	1.2	1.5	2	1.5	3	4	0.8	1	2
	FOX 4	0.3	0.4	0.5	0.4	0.8	1	0.2	0.4	1
	FOX 8	<0.016	0.03	0.13	0.03	0.2	0.25	0.016	0.08	0.25
CLR D3	-	0.2	1	2	<0.5	<0.5	<0.5	0.25	1.5	4
CLR D14	-	N.A	N.A	16	N.A	N.A	<0.5	N.A	N.A	16

^*a*^ All potency values in mg/mL. SUL is supplemented at 4 mg/mL whenever mentioned. IC_50_ and IC_90_, concentrations that inhibit 50% and 90% of growth, respectively. MIC_vis_, minimum concentration that completely inhibits growth by visual inspection 44); CLR, clarithromycin, positive control; D3, reading on day 3 as standard assay duration; D14, reading on day 14 to capture *erm41*-mediated inducible resistance in Mab subsp. *abscessus* and *bolletii*.

**Fig 2:**
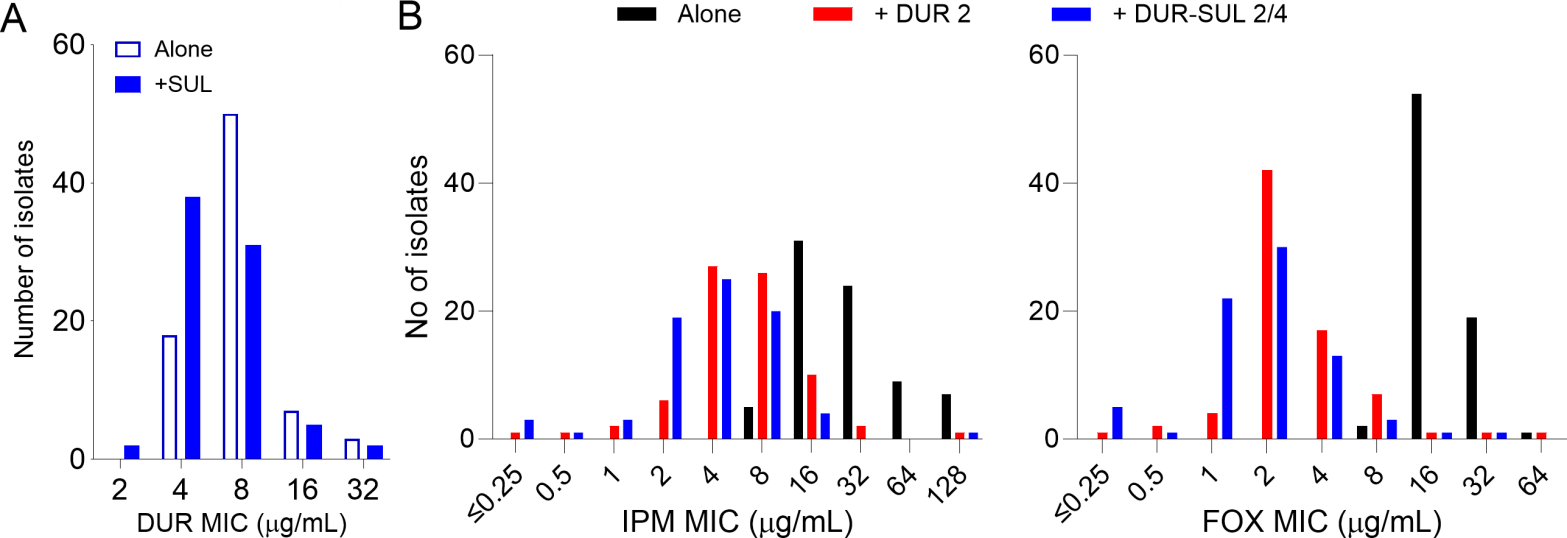
(**A**) Impact of SUL at 4 μg/mL on the MIC distribution of DUR against a panel of 72 clinical isolates representing the three subspecies of the Mab complex. (**B**) MIC distributions of IPM and FOX, in combination with DUR or DUR-SUL at the concentrations indicated (μg/mL) against 72 Mab clinical isolates: 38, 32, and 2 subsp. *abscessus*, *massiliense*, and *bolletii*, respectively, representing the frequency of clinical occurrence (46). MIC is defined as the minimum concentration inhibiting visible growth, or MIC_vis_ (44).

To quantify the impact of DUR-SUL on MIC distributions across a large panel of clinical isolates, we measured the MICs of IPM and FOX alone, and in combination with DUR 2, DUR 4, DUR-SUL 2/4, and DUR-SUL 4/4 μg/mL, against 72 isolates covering the three Mab subspecies. We found that DUR 4 and DUR-SUL 4/4 on their own inhibited growth of a substantial fraction of the isolates: 39/72 isolates susceptible to DUR-SUL 4/4 and 17/72 isolates susceptible to DUR 4. Adding SUL to DUR alone reduced the mode of DUR distribution from 8 to 4 µg/mL (Fig. 2A; Supplemental Data 1). This precluded the measurement of an MIC for IPM or FOX in combination with DUR 4 or DUR-SUL 4/4. Therefore, we combined IPM and FOX with DUR 2 and DUR-SUL 2/4 against the full panel to visualize the shift in IPM and FOX MIC distribution when potentiated by DUR (Fig. 2B; Supplemental Data 1). For IPM and FOX alone, the MIC_50_ and MIC_90_ were 32 and 64 µg/mL and 16 µg/mL and 32 µg/mL, respectively. Higher values have been reported when CAMHB is used as the growth medium and assay duration is 5 days (43, 45), as described above. Adding DUR 2 or DUR-SUL 2/4 μg/mL reduced the modes of the distribution fourfold for IPM and eightfold for FOX. Adding SUL to IPM-DUR or to FOX-DUR did not significantly shift the distribution (Fig. 2B; Fig. S2). To visualize the impact of DUR and DUR-SUL at their susceptibility breakpoints, we also plotted the IPM and FOX distributions in the presence of DUR 4 and DUR-SUL 4/4 μg/mL, resulting in effective growth inhibition of a large fraction of isolates (~25/72) at IPM and FOX concentrations ≤0.25 μg/mL (Fig. S2), driven by the lower MIC of DUR against these isolates (Supplemental Data 1).

Against Mab, the CLSI has proposed breakpoints that support susceptible (S), intermediate (I), and resistant (R) classifications as follows: S: ≤4 μg/mL, I: 8–16 μg/mL, R: ≥32 for IPM; and S: ≤16 μg/mL, I: 32–64 μg/mL, R: ≥128 for FOX. These susceptibility thresholds are higher than established for most infections (https://www.fda.gov/media/92766 [11, 47]) and are not supported by strong clinical evidence (9) due to the confounding nature of multidrug therapy. With these limitations in mind, we quantified the impact of added DUR-SUL on the susceptibility classification of the panel of clinical isolates shown in Fig. 2B. We found that adding DUR-SUL 2/4 μg/mL to IPM shifted the fraction of susceptible isolates from 0 to 68.1% (49/72 isolates) and the remaining fell in the intermediate range. For FOX, the susceptible fraction shifted from 74 to 98.6% when DUR-SUL 2/4 μg/mL was added (Table 2). Addition of DUR-SUL compared to DUR alone consistently increased the proportion of susceptible isolates. We further reasoned that the high fraction of isolates susceptible to FOX alone was partially an artifact of the very high—and seemingly arbitrary—susceptible thresholds proposed for Mab compared to Enterobacteriaceae (S ≤ 8 μg/mL), *Staphylococcus aureus* (S ≤ 4 μg/mL), or *Neisseria* (S ≤ 2 μg/mL). When we lowered the S threshold from ≤16 μg/mL to ≤8 μg/mL, the proportion of FOX-susceptible isolates increased from 3 to 97% and the 3% remaining fell in the intermediate category (Table 2). Collectively, these findings suggest the potential for enhanced clinical utility of IPM and FOX in combination with DUR-SUL against a diverse collection of Mab clinical isolates.

**TABLE 2:** Impact of DUR-SUL on the fraction of clinical isolates susceptible to IPM and FOX according to proposed susceptibility breakpoints

β-Lactams	Mab subsp. *abscessus* (*n* = 38)			Mab subsp. *massiliense* and *bolletii* (*n* = 32 + 2)			Total (*n* = 72)		
	S	I	R	S	I	R	S	I	R
IPM^*a*^	0 (0%)	23 (60.5%)	15 (39.5%)	0 (0%)	12 (35.3%)	22 (64.7)	0 (0%)	35 (47.3%)	37 (50.0%)
IPM + DUR 2 µg/mL	25 (65.8%)	12 (31.6%)	1 (2.6%)	11 (32.4%)	21 (61.8%)	2 (5.9%)	36 (50.0%)	33 (45.8%)	3 (4.7%)
IPM + DUR-SUL 2/4 µg/mL	31 (81.6%)	6 (15.8%)	1 (2.6%)	18 (52.9%)	16 (47.1%)	0 (0%)	49 (68.1%)	22 (30.5%)	1 (1.4%)
FOX high SB^*b*^	33 (86.8%)	5 (13.2%)	0 (0%)	21 (61.8%)	13 (38.2%)	0 (0%)	54 (75.0%)	18 (25.0%)	0 (0%)
FOX + DUR 2 µg/mL	37 (97.4%)	1 (2.6%)	0 (0%)	33 (97.1%)	1 (2.9%)	0 (0%)	70 (97.2%)	2 (2.8%)	0 (0%)
FOX +DUR-SUL 2/4 µg/mL	38 (100%)	0 (0%)	0 (0%)	33 (97.1%)	1 (2.9%)	0 (0%)	71 (98.6%)	1 (1.4%)	0 (0%)
FOX low SB^*c*^	2 (5.3%)	35 (92.1%)	1 (2.6%)	0 (0%)	34 (100%)	0 (0%)	2 (2.8%)	69 (95.8)	1 (1.4%)
FOX + DUR 2 µg/mL	37 (97.4%)	0 (0%)	1 (2.6%)	32 (94.1%)	2 (5.9%)	0 (0%)	69 (95.8)	2 (2.8%)	1 (1.4%)
FOX + DUR-SUL 2/4 µg/mL	37 (97.4%)	1 (2.6%)	0 (0%)	33 (97.1%)	1 (2.9%)	0 (0%)	70 (97.2%)	2 (2.8%)	0 (0%)

^*a*^ IPM, S, susceptible ≤4 μg/mL; I, intermediate 8 μg/mL–16 μg/mL; R, resistant ≥32 μg/mL.

^*b*^ FOX high susceptibility breakpoints (SB): S ≤ 16 μg/mL, I: 32 μg/mL–64 μg/mL, R ≥ 128 μg/mL.

^*c*^ FOX revised SB: S ≤ 8 μg/mL, I: 16 μg/mL–32 μg/mL, R ≥ 64 μg/mL.

### DUR-SUL enhances the bactericidal effect and bacterial cell lysis induced by IPM and FOX

**Fig 3:**
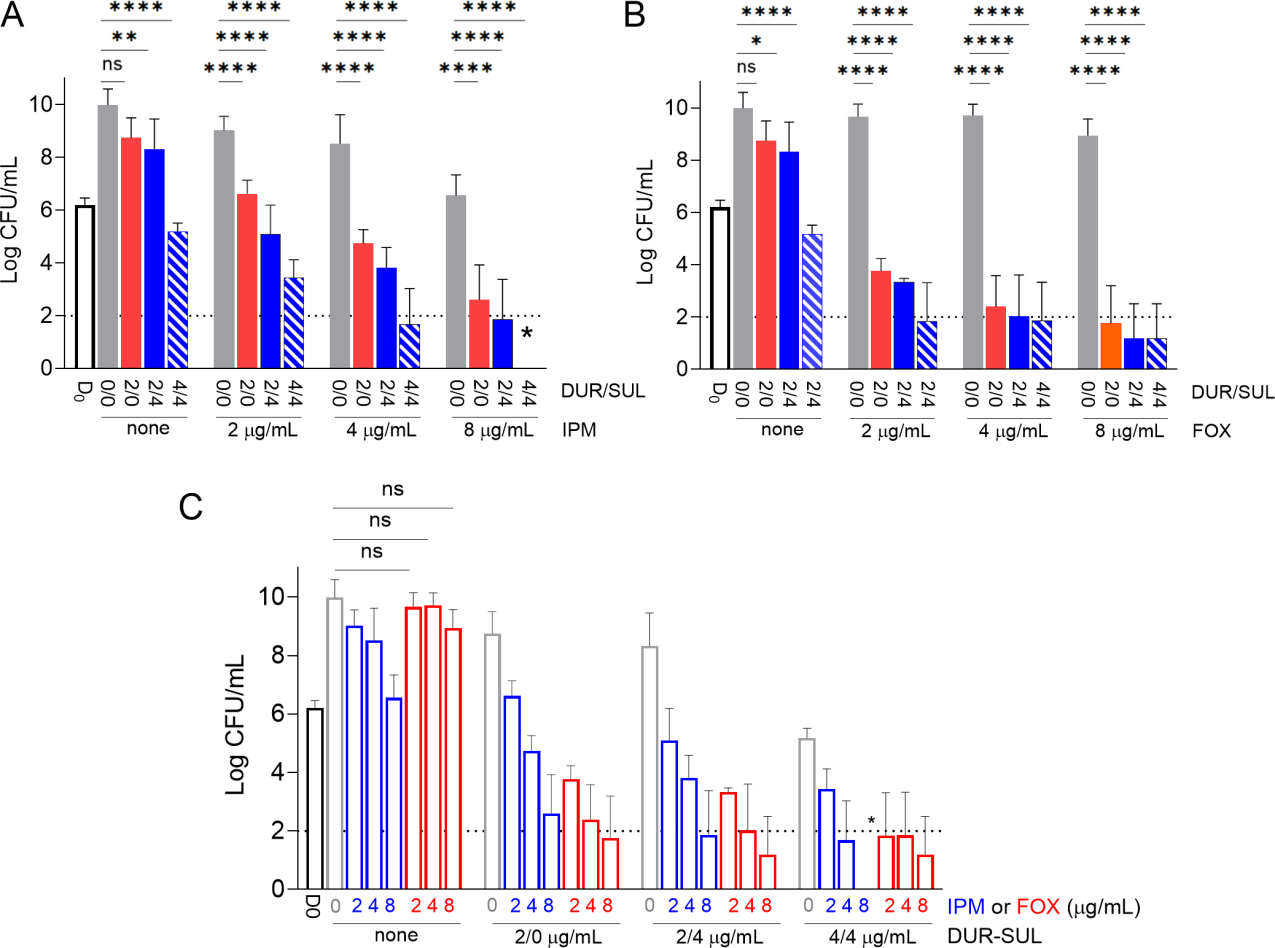
Impact of DUR-SUL and IPM or FOX on the bactericidal activity of each other, measured over 3 days. (**A**) Potentiation of IPM by DUR and DUR-SUL. (**B**) Potentiation of FOX by DUR or DUR-SUL. (**C**) Potentiation of DUR-SUL by IPM or FOX. All drug concentrations are at or below the published clinical breakpoints. The asterisk indicates that colony forming units (CFUs) were below the limit of detection (2 log, dotted line) in all replicates. Statistical analysis was performed using two-way analysis of variance with Tukey’s multiple comparison test. The statistical significance of relevant comparisons is shown. D_0_: bacterial burden prior to drug treatment. The experiment was repeated three times independently and means/standard deviations of the three data sets are shown. For clarity, only ns (not statistically significant) effects are shown in (**C**). The effects of IPM or FOX at 2, 4, and 8 μg/mL were statistically significant in all other treatment groups.* *P* < 0.05; **P < 0.01; ****P < 0.0001.

IPM and FOX, like all β-lactams, are bactericidal around their MIC (18), an important attribute in the treatment of Mab-PD since many patients suffer from systemic or localized immune deficiencies (48, 49), and the complex immunopathology promotes survival of non-replicating persisters (50). To determine whether potentiation in growth inhibition translates into enhanced bactericidal activity, we conducted concentration-kill assays in which a range of mostly sub-inhibitory IPM and FOX concentrations (2 to 8 µg/mL) were tested alone, with DUR at 2 µg/mL or with DUR-SUL at 2/4 and 4/4 µg/mL (Fig. 3A and B). As expected, IPM and FOX were not bactericidal within this concentration range. Adding DUR alone or DUR-SUL significantly potentiated both at all concentrations tested, and the effect was most pronounced on FOX, as seen in the MIC distributions. Adding DUR-SUL at 4/4 µg/mL to IPM at 4 µg/mL or FOX at 8 µg/mL, the proposed susceptibility breakpoints for each agent achieved a 4 to 5 log reduction of the initial bacterial burden over the 3-day assay duration (Fig. 3A and B; Fig. S3). Since DUR-SUL inhibits a substantial fraction of Mab clinical isolates at or below their susceptibility breakpoint (4/4), we hypothesized that DUR-SUL could be considered as the primary β-lactam injectable during the intensive phase of therapy, with IPM or FOX as potentiator for a short period to achieve rapid decrease of the bacterial burden. Under that scenario, we plotted the impact of IPM or FOX on the primary bactericidal activity of DUR and DUR-SUL. IPM or FOX at 2, 4, and 8 μg/mL significantly increased the killing achieved by DUR and DUR-SUL at all concentrations (Fig. 3C).

**Fig 4:**
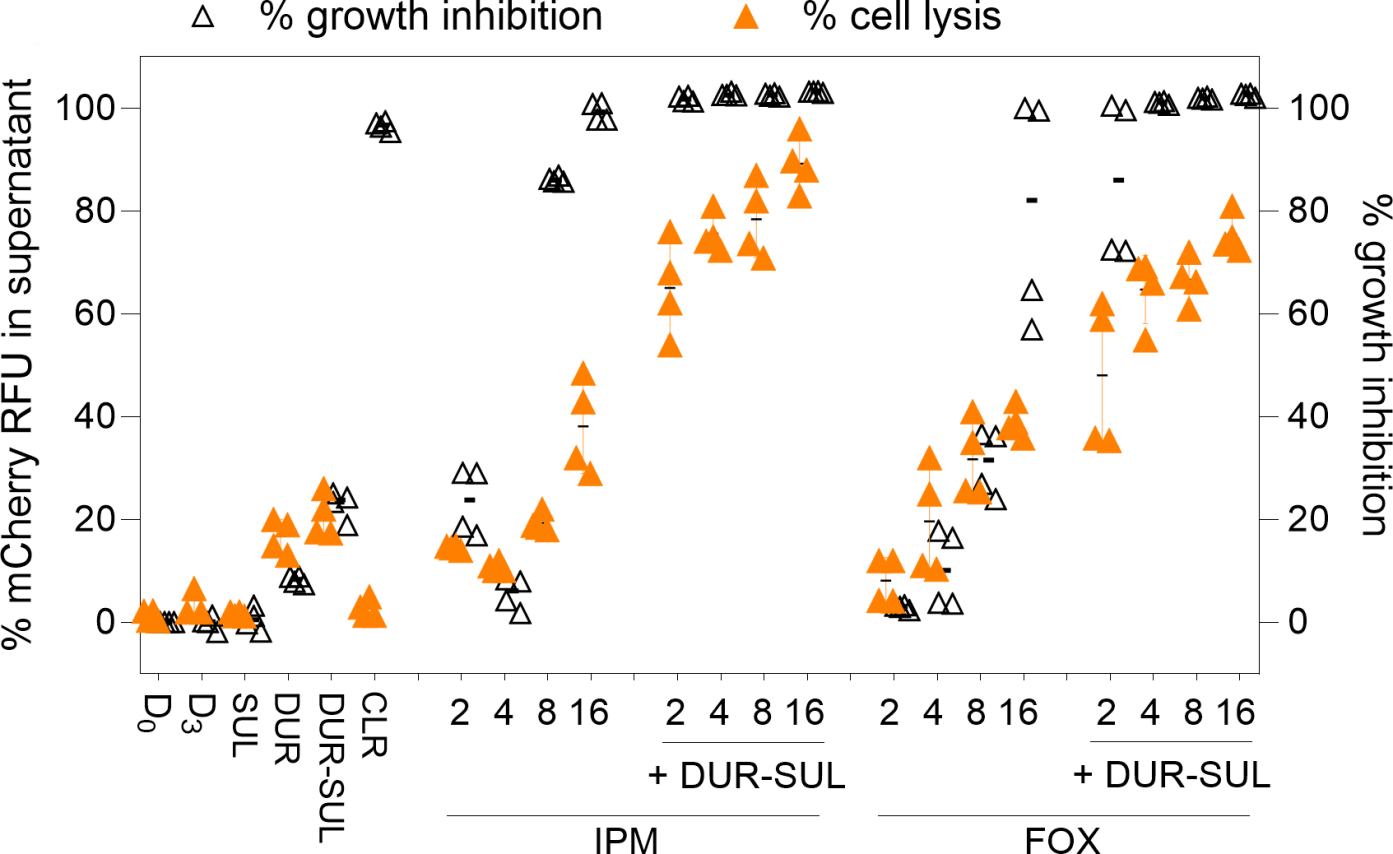
Impact of DUR-SUL on mycobacterial cell lysis induced by IPM and FOX. Mab ATCC 19977 expressing mCherry was exposed to single agents and combinations as indicated, for 3 days. DUR and/or SUL were added at 2 and 4 μg/mL, respectively. Clarithromycin (CLR), a bacteriostatic macrolide, was used as negative cell lysis control at 16 μg/mL. Growth inhibition was monitored at OD_600 nm_. The experiment was carried out twice independently in technical duplicates and all data points are shown. Data were analyzed by two-way analysis of variance and Tukey’s multiple comparison test. The addition of DUR-SUL had a positive statistically significant effect on cell lysis induced by IPM and FOX at all concentrations tested (****, *P* < 0.0001, not depicted on the graph for clarity).

Inhibition of cell wall biosynthesis by the β-lactam faropenem induces rapid cytolysis of *M. tuberculosis* single cells due to the loss of cell wall integrity, as demonstrated by quantitative time-lapse microscopy and microfluidics (51). To evaluate the impact of single and combination β-lactam treatments on the cell wall integrity of Mab, we used an mCherry fluorometric reporter (52) and monitored the release of red fluorescent protein in the culture supernatant as a readout of cell lysis. An exponentially growing culture of mCherry-Mab ATCC 19977 was exposed to 2 to 16 μg/mL of IPM or FOX, alone or with DUR-SUL at 2/4 µg/mL for 3 days. mCherry fluorescence in the supernatant was normalized to whole-culture fluorescence signal to obtain a percentage of cell lysis. At the growth inhibitory concentration of 16 µg/mL, IPM and FOX induced ~40% lysis, and DUR-SUL at 2/4 μg/mL alone caused ~20% lysis, attributable to DUR since no lysis was observed in the presence of SUL alone. Adding DUR-SUL to IPM or FOX significantly increased cell lysis, and the percentage of cell lysis induced by the combinations was more than additive at all concentrations tested (Fig. 4).

### The DUR-SUL pair suppresses emergence of resistance to IPM and FOX

To determine whether DUR-SUL has the potential to reduce the frequency of acquired resistance (FOR) to IPM and FOX, we performed a series of mutant selection experiments on solid growth medium. We first determined the agar MIC (aMIC) of DUR and DUR-SUL (8 μg/mL, with and without SUL), IPM (2 μg/mL), and FOX (32 μg/mL) (Fig. S4) and selected resistant mutants on approximately 2× and 4× aMIC. At 2× aMIC, the FORs were 7.5 × 10^−6^/CFU and 2.1 × 10^−7^/CFU for IPM and FOX, respectively. The addition of DUR-SUL at 4/4 μg/mL reduced the FOR of IPM/DUR-SUL to 1.2 × 10^−9^ and of FOX/DUR-SUL to <1 × 10^−9^ (Fig. S5). The level of IPM and FOX resistance was quantified for a representative subset of the mutants in dose-response growth inhibition assays, showing mostly twofold and occasionally fourfold increase in MIC (Table 3). To characterize the mechanism of resistance, whole genome sequencing of seven and six mutants resistant to IPM and FOX, respectively, was carried out. We found IPM-resistant mutants in *rhsA* (MAB_3542c, encoding the anti-SigH factor [53, 54]), *mspA* (MAB_1080, also referred to as *mmpA* [55] encoding a well-described porin in mycobacteria [56, 57]), and the Mab homolog of *mmpL*11 (58) (MAB_4529, encoding a putative membrane protein involved in lipid transport in *M. tuberculosis* [59]). All FOX-resistant mutants harbored mutations in *mmpL*11, or in MAB_4530, the downstream open reading frame within the same operon. In *Mycobacterium smegmatis*, the homolog of MAB_4530 (MSMEG_0240) forms an operon with *mmpL11* and is described as a transcription factor of unknown function (60). Loss-of-function mutations in either *M. smegmatis’ mmpL11* or the homolog of MAB_4530 confer resistance to a non-β-lactam putative topoisomerase inhibitor (61). Most of the mutations caused loss of function due to frameshifts. The *rshA* and *mspA* mutants retained the same susceptibility to DUR-SUL as the WT, whereas most *mmpL11* mutants displayed a slightly elevated (1.5- to 2-fold) MIC to DUR-SUL (Table 3). To confirm that these mutations were responsible for the resistance phenotype, wild-type *rshA* (MAB_3542c), *mspA* (MAB_1080), and *mmpL11* (MAB_4529) were overexpressed in the respective mutant backgrounds, and MICs were determined in liquid media, showing restoration of IPM and FOX susceptibility to the WT level (Table 4). Importantly, we also found that adding DUR-SUL to IPM or FOX suppresses the resistance phenotype of *rshA* and *mspA* mutants, but not the *mmpL11* mutants (Table 4).

**TABLE 3:** Characterization of Mab ATCC 19977 mutants resistant to IPM or FOX

Strains	IPM [μg/mL]			FOX [μg/mL]			DUR-SUL [μg/mL]			Polymorphisms and affected genes			
	IC_50_	IC_90_	MIC_Vis_	IC_50_	IC_90_	MIC_Vis_	IC_50_	IC_90_	MIC_Vis_	Gene	Gene name and protein function	Mutation	Amino acid change
WT	2.5	4	16	7	12	16	2	3	4	WT	WT	WT	WT
IPM_M1	2.5	10	32	7	10	16	2	2.5	4	MAB_3542c	*rshA*, anti-SigH sigma factor	216_217insCCCGCAAGTGC	G73fs
IPM_M6	6	12	32	7	12	16	2.2	2.5	4	MAB_3542c		T151C	Cys51Arg
IPM_M2	4	8	32	10	15	16	2	3.5	4	MAB_1080	*mspA*, porin	C200T	P67L
IPM_M3	6	12	32	9	15	16	2.5	3.5	4	MAB_1080		211dupA	R70fs
IPM_M4	4	20	64	10	12	16	2.5	3	4	MAB_1080		90_91insGC	A30fs
IPM_M5	4	32	>64	8	15	32	3.5	5	8	MAB_4529	*mmpL11,* Putative membrane protein MmpL11	G184T	E62X
IPM_M7	10	15	>64	15	16	32	4	5.5	8	MAB_4529		316dupC	I105fs
FOX_M1	6	10	32	10	15	32	2.5	3.5	4	MAB_4529	*mmpL11*, Putative membrane protein MmpL11	T2107G	W703G
FOX_M2	20	32	>64	15	20	32	3.5	5	8	MAB_4529		2119delG	V707fs
FOX_M3	20	32	>64	15	18	>32	3.5	6	8	MAB_4530		465_468del	M155fs
FOX_M4	20	40	>64	15	20	>32	3.5	6	8	MAB_4529		1783dupC	P594fs
FOX_M5	20	40	>64	15	20	>32	4	6	8	MAB_4529		G493A, 2737_2738insCGGTGG	A165T; S913delinsSVA
FOX_M6	20	40	>64	15	20	32	3.5	6	8	MAB_4529		G1472A	W491X

**TABLE 4:** Complementation of IPM- and FOX-resistant mutants^*a*^

Strains	IPM [µg/mL]		IPM [µg/mL] + DUR + SUL		FOX [µg/mL]		FOX [µg/mL] + DUR + SUL		DUR [µg/mL] + SUL	
	IC_90_	MIC_vis_	IC_90_	MIC_vis_	IC_90_	MIC_vis_	IC_90_	MIC_vis_	IC_90_	MICvis
WT Mab ATCC 19977	4	16	1.5	2.0	12	16	1.5	2.0	3.5	4
WT Mab empty vector)	5	16	1.2	2.0	12	16	1.0	2.0	3.5	4
IPM_M1 (*rshA* mutant)	12	32	1.5	2.0	12	16	0.5	1.0	2.5	3
IPM_M1 (OE *rshA*)	4	16	1.5	2.0	12	16	0.5	1.0	2.5	4
IPM_M4 (*mspA* mutant)	24	64	1.5	4.0	12	16	1.2	2.0	2.5	3
IPM_M4 (OE *mspA*)	6	16	1.0	2.0	11	16	1.0	2.0	2.5	3
FOX_M4 (*mmpL11* mutant)	40	>64	3.5	4.0	24	32	3.0	4.0	3.5	8
FOX_M4 (OE *mmpL11*)	10	32	1.5	2.0	12	16	1.5	2.0	2.5	4

^*a*^ OE, overexpression of the indicated WT gene in the resistant strain harboring the corresponding frameshift allele (Table 3).

### MspA and MmpL11 loss-of-function mutations confer cross-resistance to distinct drug classes and are found in clinical isolates

Since MspA and MmpL11 are transmembrane proteins with demonstrated and putative small molecule transport functions, respectively, we hypothesized that *mspA* and *mmpL11* loss-of-function mutations may confer cross-resistance to other antibiotics. We measured the MIC of standard of care drugs recommended for the treatment of Mab-PD against representative *mspA* and *mmpL11* mutants, as well as the complemented strains, and found that an IPM-resistant *mspA* mutant conferred low-level cross-resistance to tigecycline, and that loss of *mmpL11* in a FOX-resistant mutant conferred high-level cross-resistance to imipenem, tigecycline, and omadacycline, and low-level resistance to amikacin, moxifloxacin, and clofazimine (Table 5; Fig. S6).

**TABLE 5:** Activity (IC_90_) of clinically relevant anti-nontuberculous mycobacterial agents against IPM- and FOX-resistant mutants harboring mutations in *mspA* and *mmpL11* homolog

Drug	logP	WT Mab ATCC 19977	IPM_M4 (*mspA* mutant)	IPM_M4 (OE *mspA*)^*c*^	FOX_M4 (*mmpL11* mutant)	FOX_M4 (OE *mmpL11*)
Imipenem	−2.8	5	16	4	64	10
Amikacin	−3.3	5	7	3	16	4
Tigecycline	−1.3	7	16	8	>32	12
Omadacycline	0.4	5	7	4	>32	7
Linezolid	0.3	3	2.5	1.5	4	3.5
Fobrepodacin^*a*^	1.4	0.4	0.4	0.4	0.6	0.3
Moxifloxacin	1.6	1.5	1.2	0.8	4	1.5
Quabodepistat^*b*^	2.5	3	3.5	3	3	5
Clarithromycin	3.2	0.5	0.8	0.25	1	0.5
Rifabutin	3.5	1	1	0.8	1.5	1
Clofazimine	7.3	4	4	4	16	8
Bedaquiline	7.6	0.25	0.5	0.13	0.25	0.25

^*a*^ Gyrase B inhibitor SPR720, the prodrug of SPR719 used in the assay.

^*b*^ Not a Mab-PD drug, included as an additional cell wall biosynthesis inhibitor with a novel bactericidal mechanism of action.

^*c*^ OE, overexpression of the indicated WT gene in the resistant strain harboring the corresponding frameshift allele (Table 3).

To assess the clinical relevance of these polymorphisms, we surveyed whole genome sequences of 10,000 clinical isolates and found 40 different loss-of-function MspA mutations in 221 strains and 11 different loss-of-function MmpL11 mutations across 30 strains (Supplemental Data 2 and 3). Moreover, *mspA* was recently identified as a stepping stone resistance mutation acquired in a patient treated with IPM (62). Collectively, these results indicate that polymorphisms in *mspA* and *mmpL11* are common in clinical isolates and have the potential to confer cross-resistance to several standard of care agents, including high-level resistance by *mmpL11* to omadacycline, a new agent for the treatment of Mab-PD.

## DISCUSSION

In this study, we focused on the two β-lactam injectables recommended in the intensive treatment phase of Mab-PD and leveraged recent successes of dual β-lactam approaches to quantify the potentiation achieved by DUR-SUL—commercialized as a single formulation—added to IPM or FOX at clinically relevant concentrations. Our results also confirm the published impact of DUR on IPM’s growth inhibitory activity (32) and extend it to DUR-SUL on both IPM and FOX. Because bactericidal activity is a critical antibiotic feature to eradicate persister populations in microenvironments lacking immunity (48, 63), we verified that the synergies observed by measure of growth inhibition translated into potentiation of the β-lactams’ bactericidal and cytolytic activity by DUR-SUL. We found that the combinations bring the MICs, minimum bactericidal activities (MBCs), and cytolytic concentrations of IPM and FOX below their susceptibility breakpoints across a large panel of clinical isolates. Since DUR on its own inhibits the growth of a substantial fraction of clinical isolates at its susceptibility breakpoint of 4 μg/mL, the combinations become potent at IPM or FOX concentrations that are achieved for the entire dosing interval (0.25 μg/mL) (64, 65) with a strong positive impact on the probability of target attainment (14, 66–68) even in patients receiving IPM twice daily instead of three times due to practical considerations associated with the long treatment duration. Given the wide range of combined concentrations that achieve full growth inhibition across clinical isolates (Fig. 2; Fig. S2), drug susceptibility testing prior to treatment initiation could therefore help identify and stratify patients who would benefit the most from this treatment approach in clinical practice and clinical trials.

A major strength of the present study is the care taken to use concentration ranges aligned with clinically achieved concentrations for each agent in all experiments, for meaningful therapeutic translation. Although clinical breakpoints or susceptibility thresholds have been proposed for IPM and FOX in the context of Mab-PD, they are not convincingly supported by clinical evidence but rather seem to correspond to epidemiological cutoffs (9). Indeed, they are markedly higher than evidence-based susceptibility thresholds for other bacterial infections, leading to overestimating the susceptible fractions of clinical isolates. Under the hypothesis that clinical breakpoints rigorously established for other pathogens constitute more realistic—yet imperfect—susceptibility thresholds for Mab, adding DUR-SUL to IPM or FOX causes a marked shift from resistant to susceptible across our panel of clinical isolates. Published probabilities of target attainment (PTA) for IPM (66–68) indicate a negligible probability of achieving 40% free time above MIC (*f*T > MIC) when Mab MIC distributions of IPM alone are considered, whereas the same target of 40% *f*T/MIC has an estimated PTA > 60% when DUR-IPM MICs are considered and assuming suboptimal (but common in Mab-PD) twice-daily 1 g injections (67).

Since DUR and DUR-SUL inhibit a substantial fraction of Mab clinical isolates at or below their susceptibility breakpoint (4/4 μg/mL), either could be considered as the primary β-lactam injectable during the intensive phase, with IPM or FOX added for a short period to rapidly decrease the bacterial burden given the striking bactericidal potentiation. This new treatment paradigm could be considered in future clinical trials.

β-Lactam susceptibility is influenced by the growth medium, assay duration due to drug stability, and inoculum size (43, 69). Overall, the MIC distributions reported here for IPM and FOX as single drugs are on par with some published reports (26), and lower than others (45, 70). We attribute the differences to assay adaptations we introduced to mitigate β-lactam instability, as previously proposed by others (29).

The FOR to IPM was in line with previous reports (71). Unsurprisingly, given the functional redundancy of PBPs and peptidoglycan synthesis enzymes (24, 25), we did not identify resistance mutations in canonical β-lactam targets. Instead, we isolated IPM-resistant mutants in MspA, a porin first described in *M. smegmatis*, the loss of which confers resistance to β-lactams and other hydrophilic drug classes that appear to rely on porins for entry into mycobacteria (61, 72, 73). Genetically engineered deletion of *mspA* (also called *mmpA*) in a clinical Mab isolate conferred two- to fourfold increase in IPM MIC (55). In this work, systematic cross-resistance investigations revealed that loss of MspA only impacted the potency of tigecycline among a comprehensive set of antibiotics used to treat Mab-PD.

In a recent study of within-host evolution of drug resistance during treatment with IPM, MspA loss-of-function caused a fourfold increase in IPM MIC (8 to 32 µg/mL), and provided a stepping stone for the development of high-level (MIC >512 µg/mL) resistance conferred by mutations in an ATP-dependent helicase and increased expression of Bla_Mab_ (74). While we found *mspA* polymorphisms in a substantial fraction of clinical isolates (221/10,000), the finding that DUR-SUL abrogated resistance of *mspA* mutants to IPM and FOX dampens the clinical concerns and advocates for the use of DUR-SUL in combination with IPM or FOX.

We also isolated IPM- and FOX-resistant mutants in MmpL11, a less thoroughly characterized membrane protein that has been implicated in lipid transport, membrane permeability, survival to ubiquitin-derived peptides, and survival in macrophages (75). It is also involved in the resistance of Mab and *M. tuberculosis* to a novel topoisomerase inhibitor (61). Added DUR-SUL did not fully abrogate the resistance of *mmpL11* mutants to IPM and FOX. The multidrug cross-resistance findings combined with the presence of *mmpL11* loss-of-function mutations in a notable fraction of Mab strains suggest it may constitute another clinically relevant stepping stone toward canonical resistance acquisition.

In summary, we propose that adding DUR-SUL to first-line injectables IPM or FOX could markedly improve their clinical utility since it brings their MIC and bactericidal activity within therapeutically achieved concentrations. Although FOX is less commonly used than IPM to treat Mab-PD, the potentiation magnitude by DUR-SUL is higher for FOX than IPM, and FOX is more stable than IPM at ambient temperature, an advantage for at-home infusion in clinical practice (76).

## MATERIALS AND METHODS

### Bacterial strains, growth conditions, and chemicals

*Mycobacterium abscessus* subsp. *abscessus* ATCC 19977, harboring the inducible clarithromycin (CLR) resistance determinant *erm* (41) T28 sequevar, was purchased from the American Type Culture Collection. *M. abscessus* subsp. *bolletii* CCUG 50184T, also *erm* (41) positive, and *M. abscessus* subsp. *massiliense* CCUG 48898T, which harbors a nonfunctional *erm* (41) deletion sequevar, were purchased from the Culture Collection University of Gothenburg. The *M. abscessus* subsp. *abscessus* Bamboo strain (77) was provided by Wei Chang Huang at Taichung Veterans General Hospital, Taiwan. Mab clinical isolates were supplied by Dr. Jeanette W. P. Teo (Department of Laboratory Medicine, National University Hospital of Singapore), Yonsei University College of Medicine and Samsung Medical Center, Seoul, South Korea (78, 79). All Mab strains were cultured in complete Middlebrook 7H9 broth (271310; BD Difco, Sparks, MD, USA) supplemented with 0.05% Tween 80, 0.2% glycerol, and 10% albumin-dextrose-catalase and Middlebrook 7H10 agar (BD Difco, Sparks, MD, USA) as solid medium. BLIs were purchased from MedChemExpress LLC (USA). IPM, FOX, and CLR were purchased from Sigma-Aldrich, USA. All compounds were dissolved at 10 mM in dimethyl sulfoxide (DMSO, except for imipenem, which was dissolved in distilled water.

### Single-point screening and MIC assays

For systematic single-point screening, IPM and FOX were incubated at 10 µM in the absence and presence of BLIs at the concentrations indicated (Fig. S1; Table S1). Drugs were dispensed onto 96-well plates (Costar 3599; Corning, USA) using a TECAN D300e dispenser to achieve the desired final concentrations. Exponentially growing cultures of Mab ATCC 19977 and its isogenic Bla_Mab_ knockout strain were adjusted to a final OD_600_ of 0.005 in complete Middlebrook 7H9 broth, and 200 µL were seeded into the 96-well plates containing the dispensed drugs. The plates were sealed with parafilm and incubated at 37°C with 90 rpm shaking for 3 days. Following brief re-suspension of the cultures, absorbance was measured using a TECAN Infinite Pro 200 plate reader to calculate % growth inhibition relative to the untreated control from which the day 0 absorbance was subtracted. For MIC determinations, we followed the CLSI guidelines (44) with the following modifications to mitigate β-lactam instability in growth media (40, 80): CAMHB was substituted for Middlebrook 7H9 media in which Mab grows faster (29), and the incubation period was reduced from 5 to 3 days. Briefly, drugs were dispensed using a TECAN D300e dispenser, exponentially growing cultures of Mab-type strains and clinical isolates were adjusted to a final OD_600_ of 0.005, and 200 µL were seeded into 96-well plates, which were incubated and read as described above. For the data shown in Fig. 1 and Fig. S1, percent growth inhibition was calculated relative to untreated control, as well as after subtracting partial growth inhibition by the BLIs alone to quantify their true additive effect. In addition, the MIC_vis_ or lowest concentration at which no growth is detected by visual inspection was determined as described (44). CLR MIC was determined on day 3 and day 14 to capture *erm*41-induced resistance, as per the CLSI protocol.

### Concentration-kill experiments

In bactericidal assays, exponentially grown Mab ATCC 19977 (final OD_600_ 0.005) was exposed to sub-inhibitory concentrations of IPM and FOX (2, 4, and 8 µg/mL), in the presence of either DUR alone at 2 and 4 µg/mL or DUR + SUL at 4 µg/mL. The plates were sealed with parafilm and incubated for 3 days at 37°C with 90 rpm shaking, after which 20 µL of the cultures were transferred to round-bottom 96-well plates containing 180 μL of phosphate-buffered saline (Thermo Fisher, USA) with 0.025% Tween 80. To prevent compound carryover, serially diluted samples were plated onto 7H10 agar supplemented with 0.4% activated charcoal (Sigma-Aldrich, USA) as described previously (81). CFUs were enumerated after 5 days of incubation at 37°C. Two-way analyses of variance with Tukey’s multiple comparison test were performed (GraphPad Prism 10.2.2) to compare selected treatment groups as indicated. The assay was performed three times independently in technical duplicates. Means and standard deviations of the three data sets are shown.

### Bacterial lysis assay

To evaluate the impact of IPM and FOX, with or without DUR or DUR-SUL, on the cell wall integrity of Mab, we utilized a fluorometric reporter system (52) where mCherry is constitutively expressed in Mab ATCC 19977 under the control of the *hsp*60 promoter (82). An exponentially growing culture of the reporter strain, adjusted to a final OD_600_ of 0.2, was exposed to 2, 4, 8, or 16 µg/mL of IPM or FOX with or without DUR-SUL at 2/4 µg/mL in a total volume of 1 mL, in 14 mL round-bottom tubes. Prior to drug introduction and following a 3-day exposure period at 37°C and 120 rpm, cultures were vortexed and 200 µL was transferred onto black/clear-bottom 96-well plates (Costar 3603; Corning, USA). From the remaining culture, 500 µL was transferred into a 1.5 mL Eppendorf tube and centrifuged at 3,200 *g* for 10 minutes. Then, 400 µL of the supernatant was filtered through 0.2 μm Cytiva Whatman mini-UniPrep syringeless filters (Fisher Scientific), and 200 µL of the filtered supernatant was transferred onto black/clear bottom 96-well plates. Fluorescence from the mCherry reporter was measured at emission 587 nm/excitation 630 nm in the whole culture and the filtered supernatant. The percentage of cell lysis was determined as the ratio of mCherry fluorescence signal in the supernatant relative to the whole-culture fluorescence signal (83). The assay was performed twice independently with two technical replicates and one representative data set is shown.

### Isolation of spontaneous resistant mutants

To isolate mutants resistant to IPM and FOX with and without DUR-SUL, we first determined the aMIC on 7H10 agar. IPM and FOX were dispensed into 48-well plates at concentrations of 3.25 to 50 µM for IPM and 6.25 to 100 µM for FOX (in twofold dilutions). Then, 500 µL of molten 7H10 medium was added to the plates and mixed gently to avoid the formation of air bubbles. Next, 10 μL of exponentially grown Mab ATCC 19977 cultures, adjusted to OD_600_ of 0.005 (~ 10^4^ CFU/mL), were spotted on the agar plates. After 5 days of incubation, plates were visually inspected for growth inhibition, and aMIC was defined as the concentration that fully inhibited growth: 6.25 μM or 2 µg/mL for IPM and 50 μM or 20 µg/mL for FOX. For resistant mutant selection, exponentially grown MAB ATCC 19977 was plated on 2× and 4× aMIC of IPM and FOX alone or combined with DUR-SUL at 4/4 µg/mL. Randomly selected IPM- and FOX-resistant mutants from two independent experiments were confirmed by re-streaking on agar plates containing the drug concentrations used for the selection. The level of resistance was quantified by a dose-response growth inhibition assay in liquid broth.

### Whole-genome sequencing and complementation

Genomic DNA was extracted as described previously (84) and whole-genome re-sequencing and bioinformatics analysis were performed by Novogene Corporation Inc, USA. Sequencing data are available upon request and the GenBank accession number of the parent strain Mab ATCC 19977 is CU458896.1. Genetic polymorphisms were identified by comparison with the sequence of the reference strain. MAB_3542c, MAB_1080, and MAB_4529 frameshift mutants were complemented by overexpressing the respective wild-type gene copy under the constitutive promoter hsp60 as described previously (81). Briefly, the MAB_1080 and MAB_3542c gene sequences were amplified using forward (MAB_1080_Fw: GCGGATCCGTGGCGTGGGATACGTATTGCG; MAB_3542c_Fw: ATGACCGACGGTGAACTC) and reverse primers (MAB_1080_Rv: CGGAATTCTCAAGGCTGCGCTGACTCAGATC; MAB_3542c_Rv: CTAGGAGTTCTCGGCCCG). The PCR products of MAB_1080 and MAB_3542c were cloned into the episomal plasmid pMV262 at *Bam*HI and *Eco*RI restriction sites. The MAB_4529 gene sequence was synthesized and cloned into the pMV262 plasmid using *Bam*HI and *Hind*III restriction sites by Azenta Life Sciences, South Plainfield, NJ, USA. The sequence-confirmed plasmids were electroporated into the respective resistant mutants as described previously (81). MICs were measured in liquid medium to confirm susceptibility restoration in the complemented strains.

### Bioinformatics analysis (QC, alignment, variant calling, and annotation)

Sequence metadata were retrieved from the SRA (Sequence Read Archive) database using the SRA toolkit (version 3.1.0), with the search terms “*Mycobacteroides abscessus*” and only those entries with whole genome sequences (WGS) were included. A total of 9,865 SRA files met these criteria, and the corresponding FASTQ files were downloaded. Quality control and trimming were performed using fastp (version 0.23.4) with default parameters (85). Alignment to the reference genome (GenBank accession number: CU458896.1) was conducted using bwa-mem2 (version 2.2.1) (86) with a minimum mapping quality threshold of 60. The aligned SAM files were converted to BAM, fixed for mate information, sorted, and indexed using samtools (version 1.11) (87), and duplicates were marked using “samtools markdup.” Variant calling was performed using bcftools (version 1.20) (87) in the multiallelic caller mode with a minimum base quality of 20, a minimum coverage of 10, and a minimum proportion of reads differing from the reference of 0.9. Variants were called using “bcftools mpileup” and “bcftools call, followed by filtering with “bcftools filter” based on the specified criteria. Variant annotation was carried out using SnpEff (version 5.2) (88). Following this, SnpSift (version 5.2) (88) was employed to filter variants within the MAB_3542c gene and extract specific fields from the Variant Call Format (VCF) files. Finally, a custom Python script was used to integrate selected fields from the annotated VCF files with key fields from the SRA metadata.

## SUPPLEMENTAL MATERIAL

Data Set S1 aac.01046-24-s0001.xlsx Complete data set used to create Fig. 2 (MIC distributions).

Data Set S2 aac.01046-24-s0002.xlsx Results of the search for genetic polymorphisms in mspA (MAB_1080) in 10,000 published genomes.

Data Set S3 aac.01046-24-s0003.xlsx Results of the search for genetic polymorphisms in mmpL11 (MAB_4529) in 10,000 published genomes.

Supplemental material aac.01046-24-s0004.pdf Tables S1 to S3; Fig. S1 to S6.

ASM does not own the copyrights to Supplemental Material that may be linked to, or accessed through, an article. The authors have granted ASM a non-exclusive, world-wide license to publish the Supplemental Material files. Please contact the corresponding author directly for reuse.
