# Supplementary material for: Durlobactam to boost the clinical utility of standard of care β-lactams against Mycobacterium abscessus lung disease
Source: Antimicrob Agents Chemother. 2024 Nov 20;69(1):e01046-24. doi: 10.1128/aac.01046-24 (PMC11784023; doi:10.1128/aac.01046-24)
Supplement: Supplemental material — Tables S1 to S3; Fig. S1 to S6. [file aac.01046-24-s0004.pdf]

Durlobactam boosts the clinical utility of standard of care β-lactams against *Mycobacterium abscessus* lung disease

Dereje A. Negatu [a,b], Wassihun Wedajo Aragaw [a], Tewodros T. Gebresilase [c,d], Sindhuja Paruchuri [a], Firat Kaya [a], Shin, S.J. [e], Peter Sander [f, g], Véronique Dartois [a,g,#], and Thomas Dick [a,g, h]

[a] Center for Discovery and Innovation, Hackensack Meridian Health, Nutley, New Jersey, USA

[b] Center for Innovative Drug Development and Therapeutic Trials for Africa (CDT-Africa), Addis Ababa University, Addis Ababa, Ethiopia

[c] Armauer Hansen Research Institute (AHRI), Addis Ababa, Ethiopia

[d] Institute of Biotechnology, Addis Ababa University, Addis Ababa, Ethiopia

[e] Department of Microbiology, Graduate School of Medical Science, Brain Korea 21 Project, Yonsei University College of Medicine, Seoul, South Korea

[f] Institut für Medizinische Mikrobiologie, Universität Zürich, Gloriastrasse 30/32, 8006 Zürich, Switzerland.

[g] National Reference Center for Mycobacteria, Zurich, Switzerland. Orcid: 0000-0003-1581-0682

[h] Department of Medical Sciences, Hackensack Meridian School of Medicine, Nutley, New Jersey, USA

[i] Department of Microbiology and Immunology, Georgetown University, Washington, DC, USA

[#] Corresponding author

Running Title:

Keywords: β-lactams, *Mycobacterium abscessus*, lung infection, MspA, MmpL11, RshA, drug resistance.

Supplemental information

Avibactam (cat#HY-14879A), relebactam (cat#HY-16752), vaborbactam (cat#HY-19930), clavulanate (cat#HY-A0256B), tazobactam (cat#HY-B1418), sulbactam (cat#HY-B0334), durlobactam (cat#HY-117974A), taniborbactam (cat#HY-109124A), enmetazobactam (cat#HY-103095), zidebactam (cat#HY-120859), nacubactam (cat#HY-109008) were purchased from MedChemExpress LLC (USA). Imipenem (cat# PHR1796), cefoxitin (cat# C4786), and clarithromycin (cat#C9742) were purchased from Sigma-Aldrich, USA. All compounds were dissolved at 10 mM in DMSO, except for imipenem, which was dissolved in distilled water.

List of abbreviations

IPM	imipenem
FOX	cefoxitin
AMX	amoxicillin
AVI	avibactam
DUR	durlobactam
ZBD	zedibactam
REL	relebactam
NACU	nacubactam
CLA	clavulanic acid
SUL	sulbactam
TZB	tazobactam
VAB	vaborbactam
TANI	taniborbactam
ENM	enmetazobactam
CLR	clarithromycin
LZD	linezolid
BDQ	bedaquiline
RBT	rifabutin
OPC	quabodepistat (OPC-167832)
MXF	moxifloxacin
TGC	tigecycline
CFZ	clofazimine
AMK	amikacin
OMC	omadacycline

Supplemental Tables

Supplemental Table 1. Activity of approved or clinical development β-lactamase inhibitors against *M. abscessus* (Mab) ATCC19977

**β-lactamase inhibitor**	**Class**	**IC_{50}** (μM)	**IC_{90}** (μM)	**Mab MIC_{vis}** (μM) [a]	**Development stage and β-lactam partner**
Avibactam (AVI)	Diazabicyclo octanone (DBO)	70	>100	>100	Approved with ceftazidime
Durlobactam (DUR)		8	12	25 (7 mg/mL)	Approved with sulbactam
Zidebactam (ZDB)		75	>100	>100	Phase III with cefepime (NCT04979806)
Relebactam (REL)		>100	>100	>100	Approved with imipenem
Nacubactam (NACU)		75	>100	>100	Phase III with cefepime/aztreonam (NCT05905055)
Clavulanic acid (CLA)	β-lactam	>100	>100	>100	Approved with amoxicillin/ ticarcillin
Sulbactam (SUL)		100	>100	>100	Approved with ampicillin
Tazobactam (TZB)		25	>100	>100	Approved with piperacillin/ceftolozane
Enmetazobactam (ENM)		50	>100	>100	Phase III with cefepime (NCT03687255)
Vaborbactam (VAB)	Boronic acid	>100	>100	>100	Approved with meropenem
Taniborbactam (TANI)		>100	>100	>100	Phase III with cefepime (NCT06168734)

[a] according to the CLSI protocol (1)

Supplemental Table 2. Minimum inhibitory concentrations of IPM, FOX, and control drug AMX in the presence and absence of β-lactamase inhibitors in the WT and Bla_{Mab} KO Mab strains

BLIs	BLI Conc (mM)	AMX [ug/mL]				IPM [µg/mL]				FOX [ug/mL]			
		WT		Bla_{Mab} KO		WT		Bla_{Mab} KO		WT		Bla_{Mab} KO	
		IC_{50}	$\mathbf{IC_{90}}$	IC_{50}	$\mathbf{IC_{90}}$	IC_{50}	IC_{90}	IC_{50}	IC_{90}	IC_{50}	IC_{90}	IC_{50}	IC_{90}
Alone	-	>32	**>32**	1.0	**5.0**	2.0	6.0	1.0	5.0	8.0	14	8.0	13
AVI	10	1.0	**10**	1.0	**5.0**	2.0	6.0	1.5	6.0	8.0	15	8.0	13
	40	1.0	**10**	1.0	**6.0**	1.5	6.0	1.5	6.0	8.0	16	8.0	15
DUR [a]	2.5	0.5	**6.0**	0.5	**5.0**	1.5	2.0	1.0	2.0	2.0	5.0	2.0	4.0
	5	0.2	**5.0**	0.2	**2.5**	0.5	1.0	0.4	0.8	0.4	1.0	0.4	1.0
ZDB	10	3.0	**15**	1.0	**6.0**	1.5	5.0	1.5	5.0	7.0	14	7.0	11
	40	1.5	**12**	1.0	**5.0**	1.0	5.0	1.5	5.0	7.0	14	6.0	12
REL	10	0.8	**12**	1.0	**5.0**	1.5	6.0	1.5	5.0	8.0	15	7.0	13
	40	1.0	**10**	1.0	**5.0**	1.0	6.0	1.5	6.0	8.0	16	7.0	15
NACU	10	1.5	**12**	1.0	**5.0**	1.0	5.0	1.5	6.0	8.0	15	8.0	12
	40	1.0	**10**	1.0	**6.0**	1.0	5.0	1.5	6.0	7.0	15	7.0	12
CLA	10	5.0	**30**	1.0	**5.0**	1.0	5.0	1.5	6.0	8.0	13	7.0	10
	40	3.0	**20**	1.0	**6.0**	1.5	5.0	1.5	6.0	6.0	10	7.0	10
SUL	10	15	**>32**	1.0	**5.0**	1.5	5.0	1.5	6.0	8.0	12	8.0	10
	40	8.0	**32**	0.5	**5.0**	1.5	4.0	1.5	4.0	6.0	10	5.0	8.0
TZB	10	6.0	**25**	1.0	**5.0**	1.5	4.0	1.5	5.0	6.0	4.0	4.0	6.0
	40	1.0	**10**	0.5	**2.0**	1.5	3.0	1.5	3.0	4.0	5.0	2.5	5.0
VAB	10	2.0	**13**	0.5	**5.0**	1.5	6.0	1.5	5.0	8.0	14	7.0	13
	40	1.5	**13**	1.0	**5.0**	1.0	6.0	1.5	6.0	8.0	16	8.0	14
TANI	10	1.0	**8.0**	1.0	**6.0**	1.5	5.0	1.5	5.0	8.0	14	7.0	11
	40	1.0	**8.0**	1.0	**6.0**	1.5	6.0	1.5	6.0	8.0	16	7.0	15
ENM	10	2.0	**13**	1.0	**6.0**	1.5	5.0	1.5	5.0	5.0	10	4.0	6.0
	40	0.5	**8.0**	0.5	**4.0**	1.5	4.0	1.5	4.0	5.0	6.0	4.0	5.0

[a] at subinhibitory concentrations of 2.5 and 5 mM since higher concentrations inhibit growth (**Table S1**).

BLIs were added at 10 and 40 µM, except for DUR which was added at 2.5 and 5 µM since it is exhibits growth inhibitory activity at higher concentrations. The IC_{90} (concentration that inhibits 90% growth) of AMX shifts from >32 to 5 µg/mL in ΔBla_{Mab} as expected and highlighted. In the wild type (WT) background, all BLIs improve the potency of AMX to various extents, except SUL, as previously shown for REL, VAB, ZDB, NACU, DUR, (2-5), providing indirect evidence that these BLIs inhibit Bla_{Mab},

The lack of BLI-mediated potentiation of IPM or FOX was known for AVI, CLA, REL, VAB, ZDB, NACU, and is now extended to SUL, TZB, TANI and ENM. Since IPM and FOX are not susceptible to Bla_{Mab} hydrolysis, the results suggest that the study BLIs do not significantly inhibit an alternate b-lactamase that would hypothetically hydrolyze IPM or FOX.

In Mab ATCC19977 ΔBla_{Mab} (6), DUR, but also SUL, TZB and ENM improve the potency of FOX, which was not seen in the WT for the latter three. One possible interpretation of this observation is that SUL, TZB and ENM are susceptible to Bla_{Mab} hydrolysis and exert PBP/transpeptidase inhibition that synergizes with FOX but not IPM (**Figure S1BC**). In the case of SUL, this is consistent with PBP occupancy studies showing that it inactivates PonA2 and binds PbpA at 2 and 16 µg/mL, respectively (7).

Supplemental Table 3. Checkerboard analysis of IPM and FOX with DUR or DUR+SUL against *M. abscessus* ATCC 19977

β-lactam	MIC (or IC_{90}) [µg/mL]		FIC	FICI	Interpretation
	Alone	Combined			
IPM	16	2	0.13		
DUR	4	2	0.5	0.63	Additive
DUR+SUL	4	2	0.5		
FOX	16	4	0.25		
DUR	4	0.5	0.13	0.38	Synergistic
DUR+SUL	4	0.5	0.13		

FIC: fractional inhibitory concentration; The standard Checkerboard Titration assay was used as described previously (8). Concentration ranges were as follows: IPM and FOX 0.016 to 16 µg/mL, DUR 0.063 to 4 µg/mL, and SUL fixed at 4 µg/mL.

The FICI (fractional inhibitory concentration index) was calculated using the concentration at which at least 90% growth inhibition (IC_{90}) of the cultures was observed, as follows: ($MIC_{A\ combi}/MIC_{A\ alone}$) + ($MIC_{B\ combi}/MIC_{B\ alone}$). A FICI of < 0.5 was defined as synergy. The experiment was carried out twice yielding similar results.

Supplemental Figures

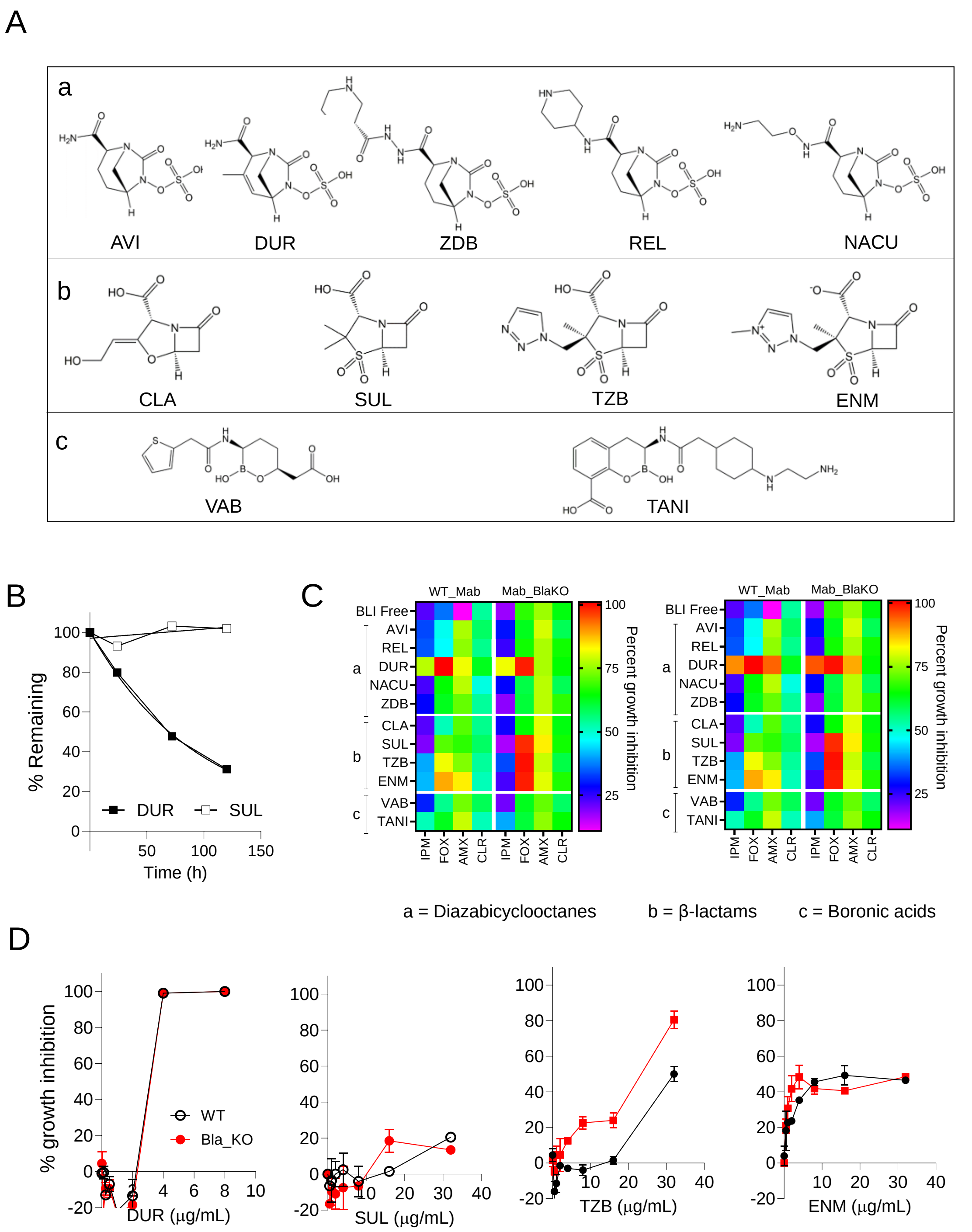

Supplemental Figure 1. Impact of β-lactamase inhibitors on the growth inhibitory activity of imipenem (IPM) and cefoxitin (FOX) against *M. abscessus* (Mab) ATCC19977. **(A)** structures of BLIs surveyed in this study; (A) Diazabicyclo[3.2.1]octanones (DBO); (B) β-lactams; (C) boronic acids. **(B)** Stability of DUR and SUL in Middlebrook 7H9 over 5 days. SUL was used as control since its stability has been published (9) and is consistent with our data. **(C)** Systematic screening of BLIs (all at 10 µM except DUR at 2.5 µM to minimize independent growth inhibition contributed by DUR) in combination with IPM and FOX at 10 µM against Mab ATCC 19977 wildtype and Bla_{Mab} knockout (KO). % growth inhibition is calculated after subtracting the growth inhibition caused by the BLI in the left panel, and without subtracting in the right panel. Amoxicillin (AMX) and clarithromycin (CLR) are included as positive and negative controls, respectively. **(D)** Dose response MIC of DUR, SUL, TZB and ENM against Mab ATCC19977 and the isogenic Bla_{Mab} KO.

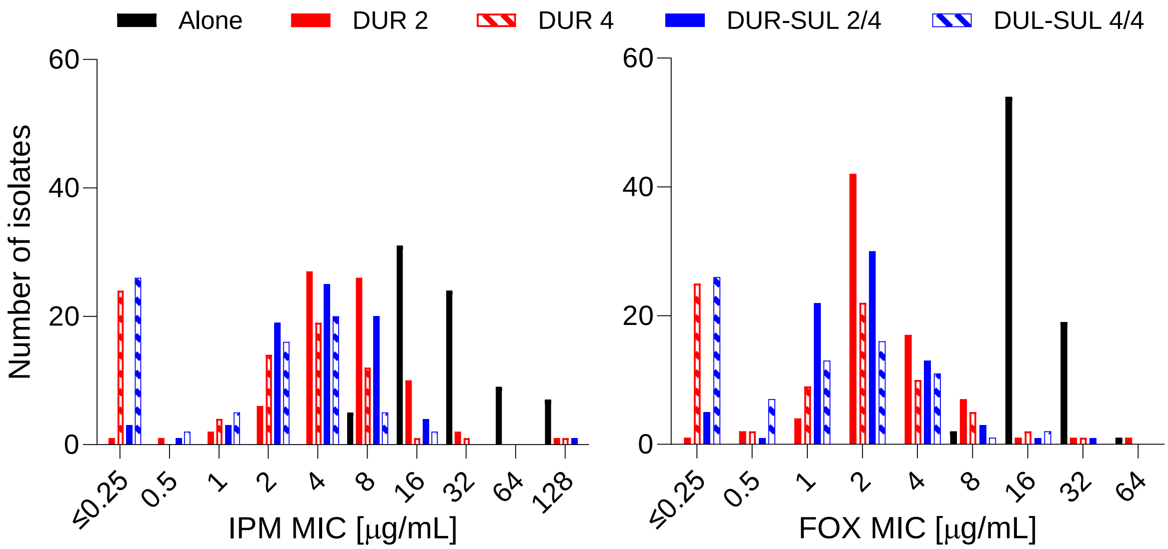

Supplemental Figure 2. MIC distributions of IPM and FOX, in combination with DUR or DUR-SUL at the concentrations indicated (µg/mL) against 72 Mab clinical isolates: 38, 32 and 2 subsp. *abscessus*, *massiliense*, and *bolletii*, respectively, representing the frequency of clinical occurrence (10). MIC is defined as the minimum concentration inhibiting visible growth, or visual MIC (MIC_{vis}) (1).

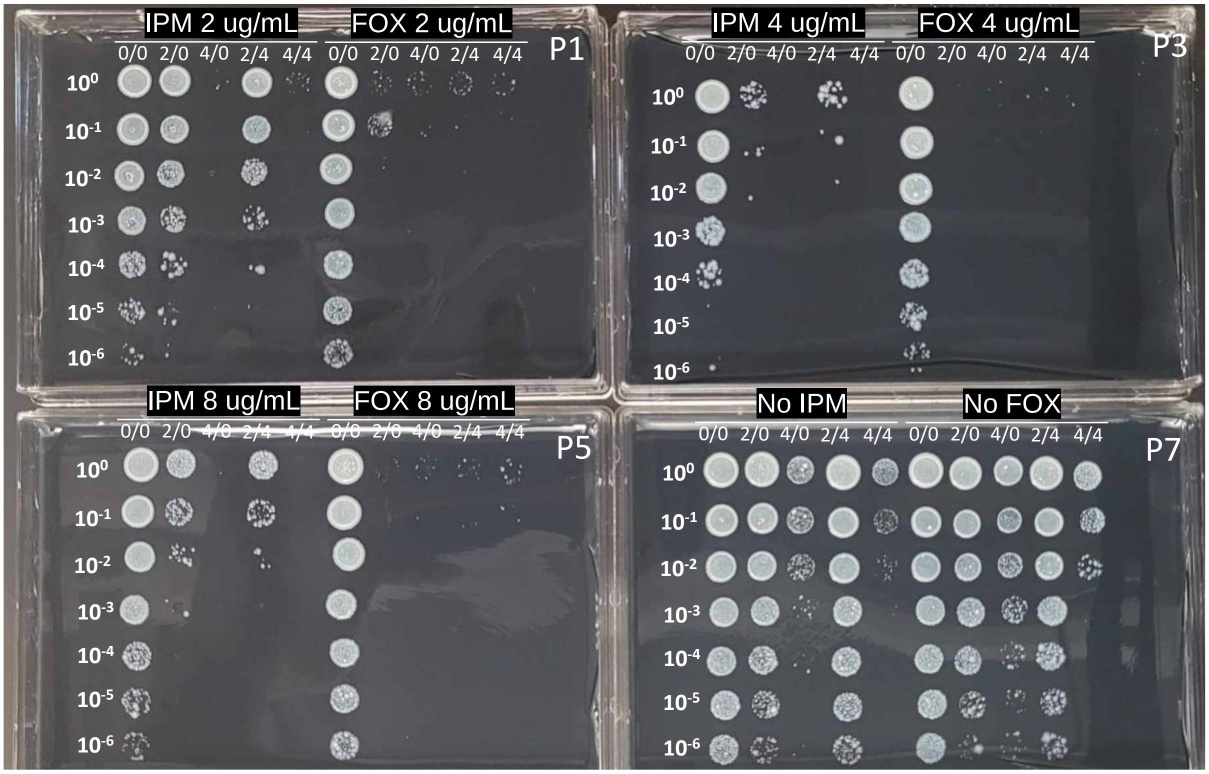

Supplemental Figure 3. Visual illustration of the impact of added DUR or DUR-SUL on the bactericidal activity of IPM and FOX. Ten-microliters of liquid cultures were spotted on Middlebrook 7H11 after 3 days of treatment, in 10-fold serial dilutions. Number pairs on top of each row indicate the concentrations of DUR-SUL used in the bactericidal assays.

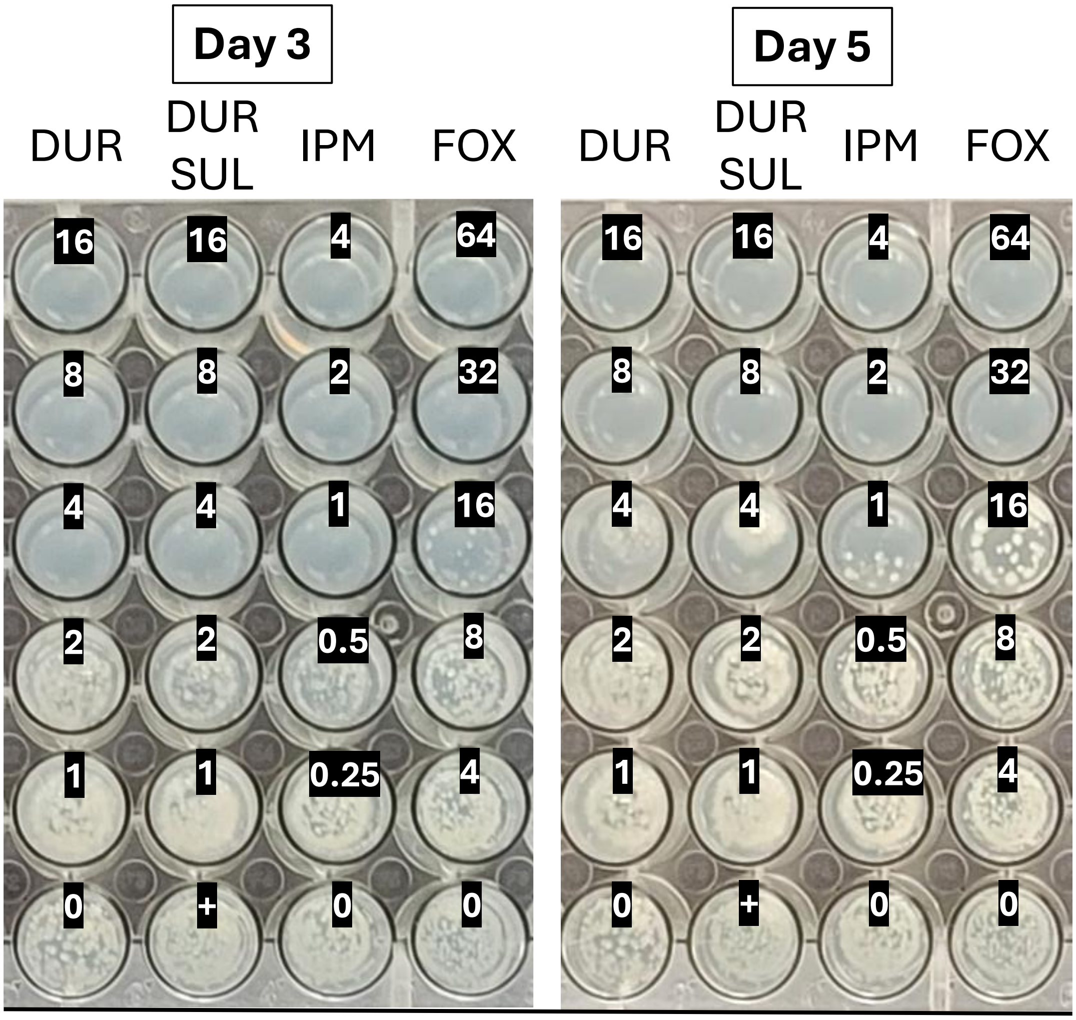

	DUR	DUR-SUL4	IPM	FOX
aMIC day 3 (µg/mL)	4	4	2	32
aMIC day 5 (µg/mL)	8	8	2	32

Supplemental Figure 4. Agar MIC (aMIC) of the study drugs, measured after 3 days (the standard MIC duration) and 5 days, the standard incubation duration to enumerate resistant mutants.

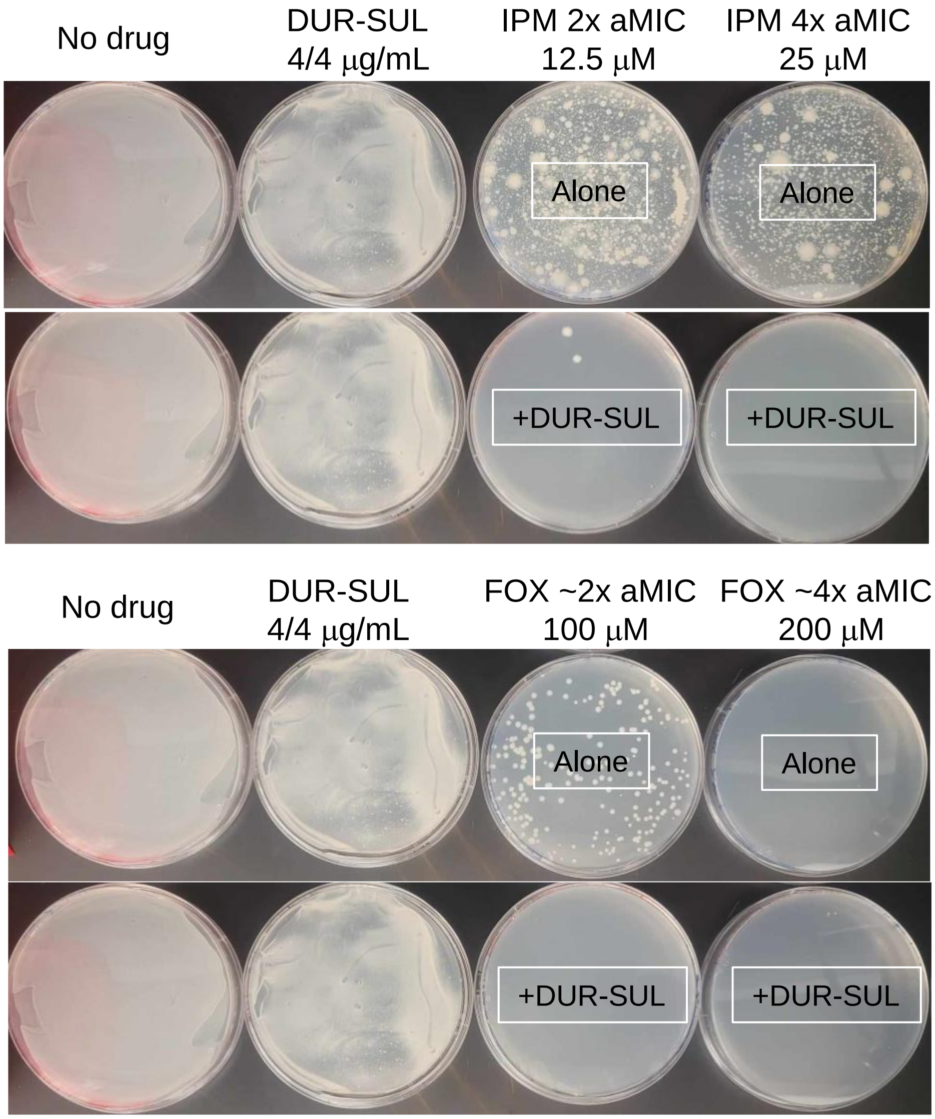

Supplemental Figure 5. Impact of DUR-SUL on the frequency of resistance to IPM and FOX. Representative examples of plates from the mutant selection experiment. Approximately 10^9 CFU were spread on each plate containing antibiotic concentrations as indicated. The heterogeneous and uniform colony morphologies observed on IPM and FOX plates, respectively, may reflect mutant diversity: in 3 different loci for IPM but all in *mmpL11* for FOX, based on WGS of selected colonies.

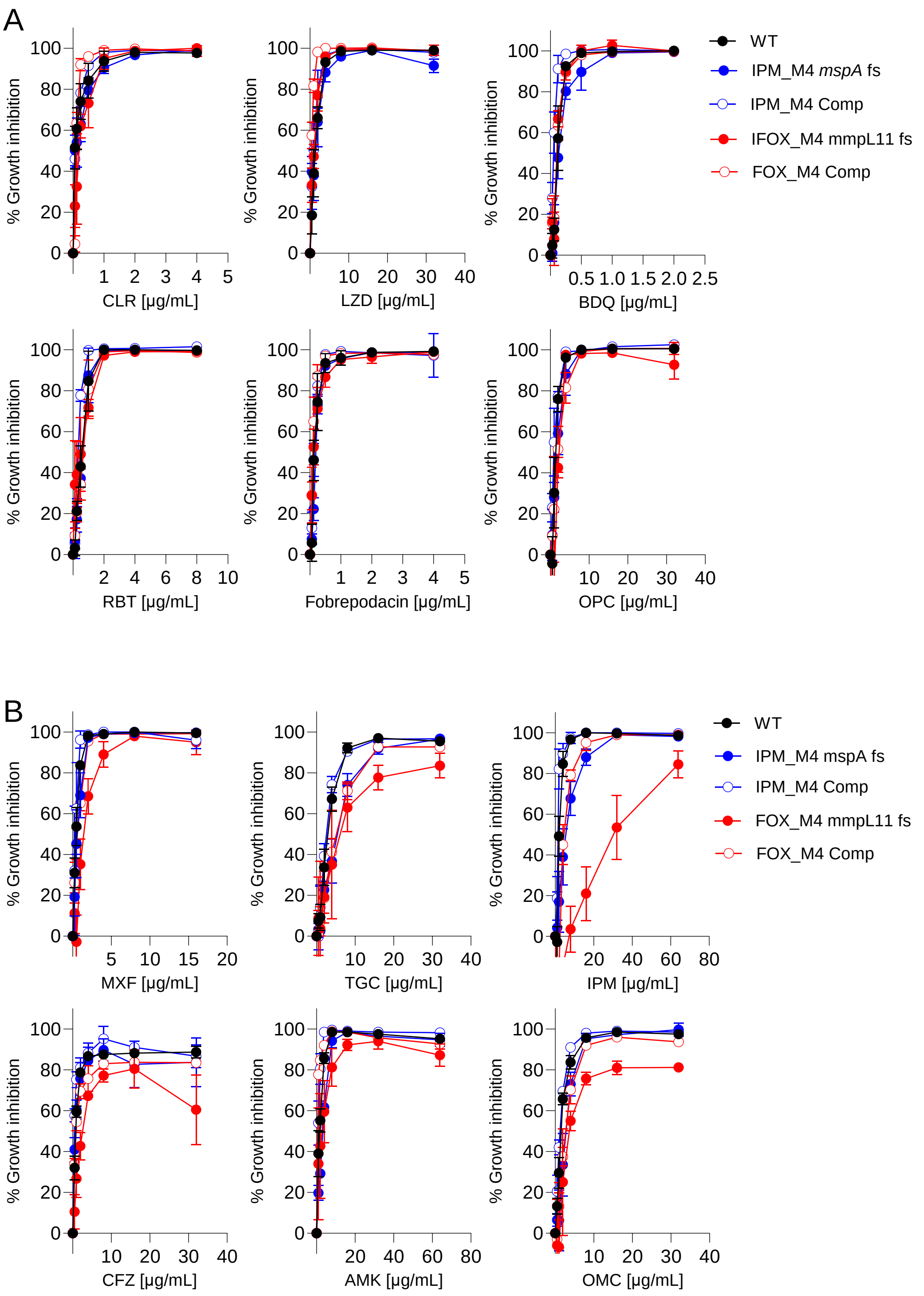

Supplemental Figure 6. Dose response MIC curves of WT ATCC19977 and the *mspA* and *mmpL11* resistant mutants isolated in the presence of IPM and FOX, respectively. The complemented strains are shown as empty symbols. **(A)** Drugs for which no cross resistance was observed. **(B)** Drugs for which deletion of either *mspA* or *mmpL11* conferred cross-resistance. Abbreviations are listed at the top of this document.

References

1. CLSI. 2018. Susceptibility testing of Mycobacteria, Nocardia spp., and Other Aerobic Actinomycetes. Clinical and Laboratory Standards Institute, Wayne, PA.
2. Lopeman RC, Harrison J, Rathbone DL, Desai M, Lambert PA, Cox JAG. 2020. Effect of Amoxicillin in combination with Imipenem-Relebactam against Mycobacterium abscessus. Sci Rep 10:928.
3. Kaushik A, Ammerman NC, Parrish NM, Nuermberger EL. 2019. New beta-Lactamase Inhibitors Nacubactam and Zidebactam Improve the In Vitro Activity of beta-Lactam Antibiotics against Mycobacterium abscessus Complex Clinical Isolates. Antimicrob Agents Chemother 63.
4. Kaushik A, Ammerman NC, Lee J, Martins O, Kreiswirth BN, Lamichhane G, Parrish NM, Nuermberger EL. 2019. In Vitro Activity of the New beta-Lactamase Inhibitors Relebactam and Vaborbactam in Combination with beta-Lactams against Mycobacterium abscessus Complex Clinical Isolates. Antimicrob Agents Chemother 63.
5. Dousa KM, Nguyen DC, Kurz SG, Taracila MA, Bethel CR, Schinabeck W, Kreiswirth BN, Brown ST, Boom WH, Hotchkiss RS, Remy KE, Jacono FJ, Daley CL, Holland SM, Miller AA, Bonomo RA. 2022. Inhibiting Mycobacterium abscessus Cell Wall Synthesis: Using a Novel Diazabicyclooctane beta-Lactamase Inhibitor To Augment beta-Lactam Action. mBio doi:10.1128/mbio.03529-21:e0352921.
6. Rominski A, Schulthess B, Muller DM, Keller PM, Sander P. 2017. Effect of beta-lactamase production and beta-lactam instability on MIC testing results for Mycobacterium abscessus. J Antimicrob Chemother 72:3070-3078.
7. Sayed ARM, Shah NR, Basso KB, Kamat M, Jiao Y, Moya B, Sutaria DS, Lang Y, Tao X, Liu W, Shin E, Zhou J, Werkman C, Louie A, Drusano GL, Bulitta JB. 2020. First Penicillin-Binding Protein Occupancy Patterns for 15 beta-Lactams and beta-Lactamase Inhibitors in Mycobacterium abscessus. Antimicrob Agents Chemother 65.
8. Negatu DA, Zimmerman MD, Dartois V, Dick T. 2022. Strongly Bactericidal All-Oral beta-Lactam Combinations for the Treatment of Mycobacterium abscessus Lung Disease. Antimicrob Agents Chemother 66:e0079022.
9. Zhou J, Qian Y, Lang Y, Zhang Y, Tao X, Moya B, Sayed ARM, Landersdorfer CB, Shin E, Werkman C, Smith NM, Kim TH, Kumaraswamy M, Shin BS, Tsuji BT, Bonomo RA, Lee RE, Bulitta JB. 2024. Comprehensive stability analysis of 13 beta-lactams and beta-lactamase inhibitors in in vitro media, and novel supplement dosing strategy to mitigate thermal drug degradation. Antimicrob Agents Chemother 68:e0139923.
10. Cristancho-Rojas C, Varley CD, Lara SC, Kherabi Y, Henkle E, Winthrop KL. 2023. Epidemiology of Mycobacterium abscessus. Clin Microbiol Infect doi:10.1016/j.cmi.2023.08.035.
